# d_*z*^2^_ Band Links Frontier Orbitals and Charge Carrier Dynamics of Single-Atom Cocatalyst-Aided Photocatalytic H_2_ Production

**DOI:** 10.1021/jacs.3c10661

**Published:** 2023-12-12

**Authors:** Yiwei Fu, Kejian Lu, Anlan Hu, Jie Huang, Liejin Guo, Jian Zhou, Jin Zhao, Oleg V. Prezhdo, Maochang Liu

**Affiliations:** ⊗ International Research Center for Renewable Energy, State Key Laboratory of Multiphase Flow, Xi′an Jiaotong University, Xi′an, Shaanxi 710049, P. R. China; ‡ Center for Alloy Innovation and Design, State Key Laboratory for Mechanical Behavior of Materials, School of Materials Science and Engineering, Xi′an Jiaotong University, Xi′an, Shaanxi 710049, P. R. China; § ICQD/Hefei National Laboratory for Physical Sciences at the Microscale, CAS Key Laboratory of Strongly-Coupled Quantum Matter Physics, and Department of Physics, University of Science and Technology of China, Hefei, Anhui 230026, P. R. China; ∥ Synergetic Innovation Center of Quantum Information & Quantum Physics, University of Science and Technology of China, Hefei, Anhui 230026, P. R. China; ⊥ Department of Chemistry and Department of Physics and Astronomy, University of Southern California, Los Angeles, California 90089, United States; # Suzhou Academy of Xi′an Jiaotong University, Suzhou, Jiangsu 215123, P. R. China

## Abstract

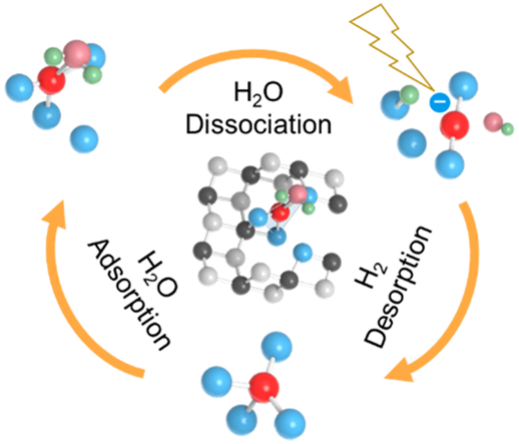

The Cu single-atom catalyst (SAC) supported on TiO_2_ exhibits outstanding efficacy in photocatalytic hydrogen evolution. The precise operational mechanism remains a subject of ongoing debate. The focus resides with the interplay linking heightened catalytic activity, dynamic valence state alterations of Cu atoms, and their hybridization with H_2_O orbitals, manifested in catalyst color changes. Taking anatase TiO_2_ (101) as a prototypical surface, we perform ab initio quantum dynamics simulation to reveal that the high activity of the Cu-SAC is due to the quasi-planar coordination structure of the Cu atom after H_2_O adsorption, allowing it to trap photoexcited hot electrons and inject them into the hybridized orbital between Cu and H_2_O. The observed alterations in the valence state and the coloration can be attributed to the H atom released during H_2_O dissociation and adsorbed onto the lattice O atom neighboring the Cu-SAC. Notably, this adsorption of H atoms puts the Cu-SAC into an inert state, as opposed to an activating effect reported previously. Our work clarifies the relationship between the high photocatalytic activity and the local dynamic atomic coordination structure, providing atomistic insights into the structural changes occurring during photocatalytic reactions on SACs.

## Introduction

Photocatalytic hydrogen evolution has the potential to help reduce the use of fossil fuels and prevent global warming,^1^ but its application is limited by the low energy conversion efficiency.^2^ Loading single-atom catalysts (SACs) on metal oxides^3,4^ has proven to be a promising approach to maximize the atom utilization percentage and provides an opportunity to learn details of the mechanisms of photocatalytic reactions, especially the role of local atomic configurations.^5−11^ The reason for the high activity of SACs in photoelectrocatalysis has been studied mainly from the following two aspects:^12^ (a) unsaturated active sites enhance adsorption and activation of molecules;^13^ (b) isolated metal sites induce electron trap states.^14^ While the frontier orbital theory, or orbital-dependent d-band center theory for SACs,^15^ commonly explains the chemical adsorption and activity, the mechanism behind the effective electron trapping by isolated metal sites remains unclear, despite the recognized importance of electron trap states in SAC experiments.^16−19^ To investigate the photocatalytic mechanism, we take the example of Cu-loaded anatase TiO_2_. Our findings reveal a connection between the frontier orbital theory and the electron trap states, both associated with the d_*z*^2^_ orbital of the Cu atom. Adjusting the coordination environment, for instance, by introducing oxygen vacancies or adsorbing small molecules, allows control of the energy and occupancy of the d_*z*^2^_ orbital, in turn influencing the activity of SACs during chemical reactions.

Despite years of research, the mechanism of the photocatalytic water dissociation on TiO_2_ is still under investigation.^20^ Among the various SACs supported on TiO_2_, Cu, an abundant transition metal, exhibits substantial photocatalytic activity comparable to that of noble metals such as Pt, Au, and Pd. Notably, Cu-SAC/TiO_2_ demonstrates intriguingly reversible alterations in color and dynamic changes in valence throughout the photocatalytic reaction.^21,22^ Gaining insight into the interrelation among the photocatalytic activity, reversible valence state variation, and color transformation can shed light on the factors contributing to the good performance of Cu-SAC/TiO_2_.

The pioneering investigations on Cu-SAC/TiO_2_ have primarily focused on the empirical exploration of the correlation between changes in the valence state and heightened activity.^21,22^ However, in the absence of a comprehensive theoretical analysis, the proposed reaction mechanisms cannot explain all the experimental data. For instance, queries persist concerning the gradual shift of Cu valence from Cu^2+^ to Cu^+^ over an extended period, as opposed to an abrupt transition within a brief interval,^22^ and the rationale behind the Cu_2_O post-reaction presence indicated by X-ray absorption near-edge structure (XANES) spectra, with the proposed model lacking an explicit explanation for the transformation of the coordination structure from CuO to Cu_2_O.^21^ Of significance is the definitive correlation between Cu^+^ attributes and photocatalytic activity, given the conflicting viewpoints delineated within the pioneering literature.^21,22^ Kim et al.^21^ proposed that Cu^2+^ transforms into Cu^+^ due to H atom adsorption on a neighboring lattice O atom and Cu^+^ works as a metalloenzyme, helping in the activation of adjacent Ti atoms. Previous theoretical investigations^23^ have proposed a mechanism rationalizing how Cu^+^ may contribute to the extension of the hot electron lifetime, thereby supporting the role of Cu^+^ as suggested by Kim et al. Subsequently, novel experimental findings have emerged, affording direct substantiation of charge accumulation on Cu atoms during photocatalysis.^22^ Using ultrafast absorption spectroscopy, Tang et al.^22^ unveiled the potential of Cu-SAC to capture electrons, thereby supporting the perspective that Cu^+^ functions directly as an active site. This development has inspired us to reevaluate the preceding analyses and to model the photocatalytic dissociation of H_2_O using advanced theoretical methodologies.^24^

In this work, reevaluating the previous analysis,^23^ we demonstrate that an oxygen vacancy is key for high photocatalytic activity and that the local Cu-SAC coordination structure is important for photocatalysis. We combine experiments and quantum dynamics simulations to identify the active site as the four-coordinated Cu-SAC on the TiO_2_ (101) surface with a neighboring oxygen vacancy. The quasi-planar quadrilateral structure of the Cu–O coordination is capable of trapping hot electrons through the Cu d_*z*^2^_ orbital, leading to high activity. The dynamic changes in the valence state and color can be attributed to adsorption of an H atom onto a bridging O atom neighboring Cu-SAC, with the H atom originating from the Volmer water splitting. We clarify that the ability of Cu-SAC to trap electrons is not harmful. Instead, it is the reason for the high activity. Importantly, the adsorbed H atom passivates Cu-SAC/TiO_2_ rather than activates it, indicating that the experimentally detected changes in the valence state and color are indicators of the high photocatalytic activity, rather than the origin. The insights obtained in this work rationalize the experimental results and provide comprehensive atomistic insights into the Cu-SAC/TiO_2_ photocatalytic process.

## Results

Before discussing the details of our analysis, we present an overview of the complete process of H_2_O dissociation on Cu-SAC/TiO_2_.

**Figure 1:**
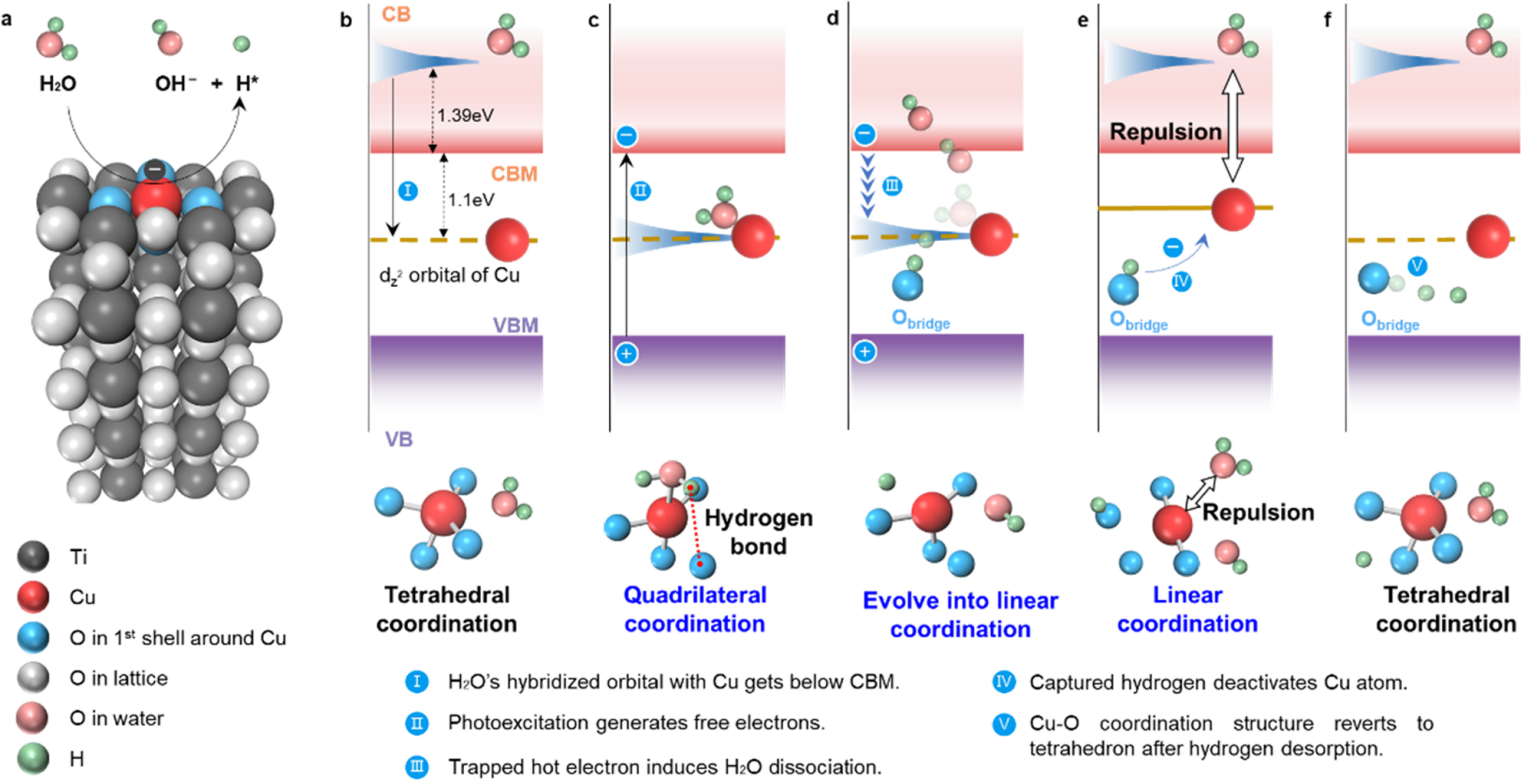
**Diagram of photocatalytic hydrogen evolution on the Cu-SAC/TiO**_**2**_**surface.** Schematics showing (a) the water splitting on the Cu-SAC/TiO_2_ (101) surface and (b–f) the steps of hydrogen evolution with the corresponding local coordination structures. The process of H_2_O splitting on Cu-SAC starts with the adsorption of H_2_O molecules onto the Cu atom. Subsequently, (b, c) a quasi-planar quadrilateral Cu–O coordination structure is formed due to hybridization of the Cu and H_2_O orbitals below the CBM of TiO_2_ and hydrogen bonding between H_2_O and lattice O atoms. (d) Under illumination, photoexcited electrons accumulate at the CBM before transition to the hybridized bandgap state formed by the hybridized d orbital of the Cu atom and p orbital of the O atom. The hot electrons trapped in the bandgap state facilitate the transfer of charge to H_2_O through the Cu–O bond, leading to H_2_O dissociation. (e) The H atoms released from the H_2_O dissociation are captured by lattice O atoms, causing a transformation of the Cu–O coordination structure into a linear arrangement. The adsorption of H atoms induces a repulsive force on subsequent H_2_O molecules, rendering the Cu-SAC inert. (f) Following H atom desorption, the Cu–O coordination structure can revert to its initial state, initiating a new cycle of photocatalysis.

Figure 1 illustrates schematically the reaction mechanism stemming from our analysis of photocatalytic H_2_O dissociation on the Cu-SAC/TiO_2_ (101) surface. Before the reaction, Cu-SAC exhibits a tetrahedral coordination configuration (Figure 1a,b). After H_2_O molecule adsorption onto the Cu-SAC, the d_*z*^2^_ orbital of the Cu-SAC hybridizes with H_2_O, creating an electron trap state below the conduction band minimum (CBM) of TiO_2_ (Figure 1b). The hybridization, together with hydrogen bonding between H atoms and lattice O atoms, produces a quasi-planar quadrilateral CuO structure (Figure 1c). Under illumination conditions, photoexcited electrons are captured by the d_*z*^2^_ orbital of the Cu-SAC and transferred to H_2_O, initiating dissociation of H_2_O into H and OH^–^ (Figure 1d). Should the released H atom from the H_2_O dissociation be captured by a neighboring bridging O atom, the carried electron is liberated from the H atom and directed into the d_*z*^2^_ orbital of the Cu-SAC (Figure 1e). The H adsorption induces a transformation from the quadrilateral Cu-SAC structure to a double-coordinated linear Cu_2_O configuration. In this instance, the Cu-SAC achieves coordination saturation, manifesting repulsiveness toward subsequent H_2_O molecules, thereby impeding further H_2_O adsorption. After H desorption (Figure 1f), the coordination structure of Cu-SAC reverts to its original tetrahedral state. This mechanism underscores that the pronounced activity of Cu-SAC/TiO_2_ arises from the quasi-planar quadrilateral Cu–O coordination conducive to H_2_O adsorption, and the subsequent harnessing of photogenerated electrons by the d_*z*^2^_ orbital of Cu, facilitating the hot electron transfer into the H_2_O molecule.

### Local Coordination Configuration of the Cu-SAC

Our analysis starts with local structural determination through computational modeling. Prior theoretical and experimental investigations have studied the coordination structures of single metal atoms on TiO_2._^7,9^ As widely accepted, SACs exhibit high reduction activity and stability upon substituting the six-coordinated Ti atom (M-Ti_6c_) or five-coordinated Ti atom (M-Ti_5c_). Extensive evidence underscores the pivotal role of Ti_5c_ as an active reduction site on a pure TiO_2_ surface, suggesting that M-Ti_5c_ may serve as an effective reaction site. This proposition is supported further by the ligand-field theory, as demonstrated in Figure S1. The d orbitals of SACs engaged in H_2_O bonding introduce bandgap states that hybridize with H_2_O orbitals, thereby fostering transfer of hot electrons photogenerated inside TiO_2_ to H_2_O. To substantiate this fact, we offer the example of the Pt-SAC. The electronic configurations of H_2_O adsorbed on Pt-Ti_6c_ and Pt-Ti_5c_ are illustrated in Figure S2. The projected density of states (PDOS) shows hybridization of the d orbitals of Cu and the 2p orbital of O in the adsorbed H_2_O. In pure TiO_2_, H_2_O adsorbs onto and hybridizes with Ti_5c_, and the Ti_5c_/H_2_O hybridized orbitals reside above the TiO_2_ CBM. Analogously, when Pt substitutes Ti_6c_ (Pt-Ti_6c_), the most favorable site for H_2_O adsorption remains Ti_5c_ due to coordination saturation of Pt-Ti_6c_. However, in the scenario of Pt replacing Ti_5c_ (Pt-Ti_5c_), H_2_O preferentially adsorbs on the Pt atom, the 2p orbital of the O atom of the H_2_O molecule hybridizes with the d orbitals of the Pt atom, and the hybridized orbitals are located below the TiO_2_ CBM. Importantly, these findings can be extrapolated to other transition metal SACs, given the universality of the ligand-field theory in the transition metal context. In order to mitigate potential inaccuracies arising from the use of DFT in describing the hybridized orbital of H_2_O adsorbed on the SACs, we compare DFT with the DFT+U method, in which a Hubbard U correction is added on the transition metal atom, as shown in Figure S3. It is observed that the relative energy difference between the hybridized orbital involving H_2_O and the TiO_2_ CBM remains relatively consistent in the two methods (see Supporting Information for details).

**Figure 2:**
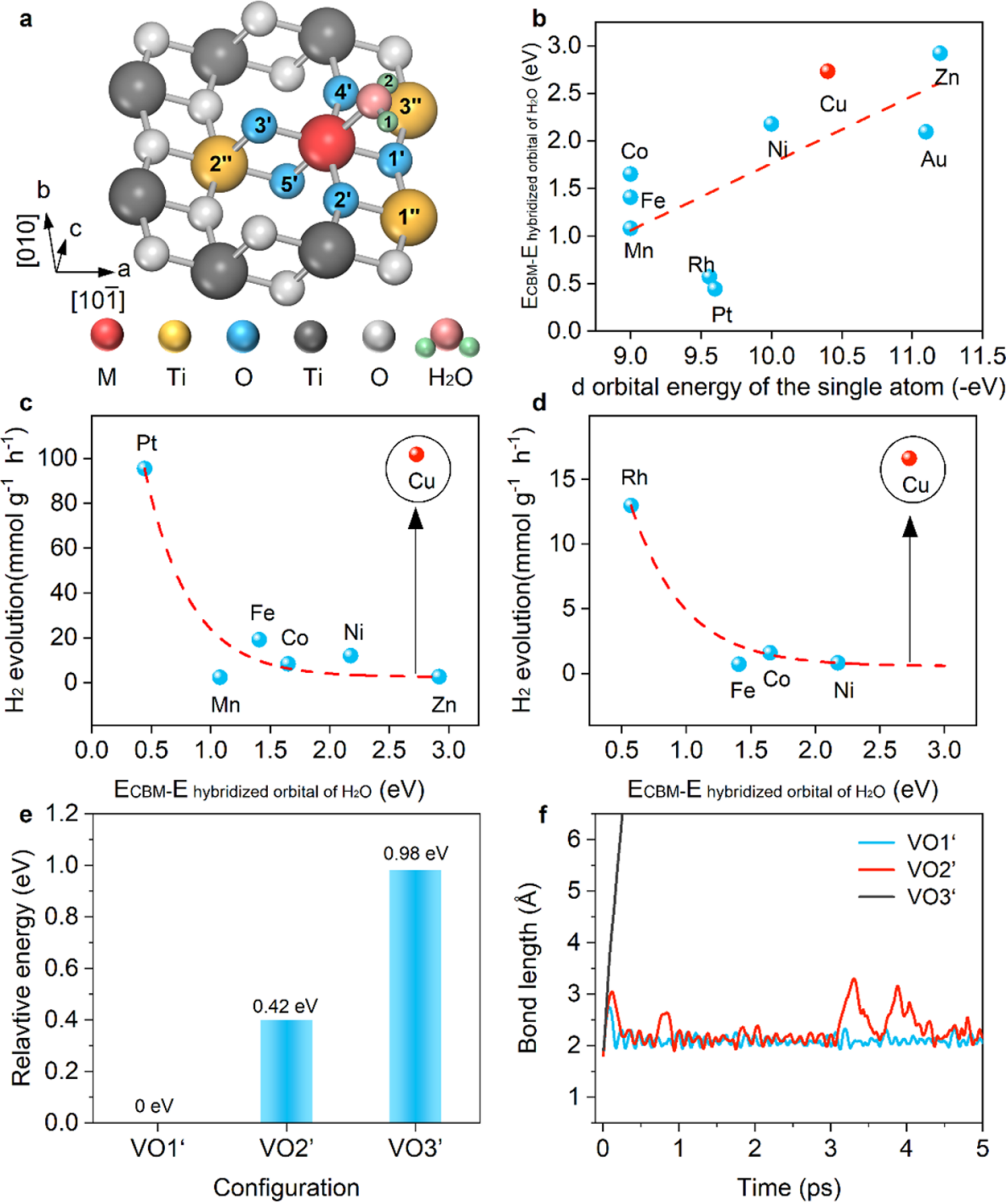
**Determination of the local coordination structure of the metal-SAC/TiO**_**2**_**.** (a) Single transition metal atom substituting Ti_5c_ on the TiO_2_ anatase (101) surface. The H atoms in the H_2_O molecule are marked as 1–2, the lattice oxygen atoms around the transition metal atom are marked as 1′–5′, and the lattice Ti atoms around the transition metal atom are marked as 1″–3″. (b) Hybridized orbital energy level of adsorbed H_2_O vs d orbital energy of different metal atoms. (c, d) Photocatalytic hydrogen evolution activity of different Metal-SACs/TiO_2_. The hydrogen evolution rates are from the experiments reported in refs (22) and (21), respectively. (e) Relative formation energies of different O atom vacancy configurations with H_2_O adsorbed on Cu-SAC. (f) Lengths of bonds between the Cu atom and the O atom in the adsorbed H_2_O molecule.

According to ligand-field theory, the e_g*_ orbital of the transition metal atom, localized within the bandgap and hybridizing with H_2_O can shift in energy for different transition metal species.^25−27^ The structure involving a transition metal SAC substituting the Ti_5c_ position (M-Ti_5c_) is employed as an example (Figure 2a). The energy levels of the hybridized orbitals are calculated, showing a positive correlation with the energies of the d orbitals of neutral metal atoms, as illustrated in Figure 2b. The d orbital energies of different transition metals, Figure S4a, are drawn from literature sources based on photoelectron spectroscopy experiments.^28^ To compare whether different data sources consistently reflect the positive correlation between the energies of the hybridized orbitals involving H_2_O and the d orbitals of the adsorbed SACs, we utilized photoelectron spectroscopy data from NIST, atomic electronic structure calculation reference data from NIST, and the current VASP calculations. The results are shown in Figure S4b–d. All data show a positive correlation between the energies of the hybridized orbital and the d orbital, indicating that if the energy of the d orbital of a SAC is inside the TiO_2_ bandgap (Figure S1) the hybridized orbital involving H_2_O is also inside the bandgap, facilitating H_2_O adsorption and hot electron capture and transfer from TiO_2_ to H_2_O.

Analysis of the photocatalytic activity of various metal SACs, Figure 2c and Figure 2d, illustrates a general trend wherein the activity diminishes as the energy level of the hybridized orbital of H_2_O decreases, with Cu-SAC being an exception. To understand this anomaly, we investigate the electronic structure of different SACs (Figure S5). The orbital hybridized between the Cu-SAC and of H_2_O lies close to the valence band maximum (VBM) of Cu-SAC/TiO_2_, which should impede hot electron transfer into H_2_O. Moreover, the hot electrons captured by the hybridized orbital fail to reach the threshold energy needed for an effective dissociation, owing to their relatively low energy levels. Curiously, despite these considerations, the photocatalytic experiments underscore Cu-SAC/TiO_2_ as an efficient catalyst for water-splitting hydrogen production. This observation signifies that the increase in the energy of the hybridized orbital during the reaction prevails over the previously stated hindrances. Notably, the increase in energy of the hybridized orbital can be realized through introduction of an oxygen vacancy around the Cu atom, which can introduce extra electrons and increase the energy of the empty d orbital of the Cu atom. Indeed, existing literature substantiates that a Cu atom on TiO_2_ can induce formation of lattice oxygen vacancies in Cu-SAC/TiO_2._^29−33^ To corroborate the above arguments, we calculate the free energy of H_2_O adsorption (Figure S6a). The results indicate that Cu-Ti_5c_ fails to stably adsorb H_2_O in the absence of an O vacancy. Additionally, microcanonical ab initio molecular dynamics (AIMD) simulations indicate that an H_2_O molecule adsorbed on Cu-Ti_5c_ desorbs within 500 fs (Figure S6b).

To elucidate the precise coordination structure of the Cu-SAC, we conducted microcanonical AIMD simulations with the initial temperature of 300 K to assess the Cu-SAC capability in adsorbing H_2_O molecules. We consider an oxygen vacancy within the first coordination shell surrounding the Cu atom. Potential surface vacancy sites are denoted as 1′–4′ in Figure 2a. Since VO2′ and VO4′ are structurally equivalent, we focus on VO2′ only. As illustrated in Figure 2e,f, only VO1′ can stably adsorb H_2_O, forming a quasi-planar configuration, as depicted in Figure 1c. Similarly, a quasi-planar arrangement between the Cu atom and its surrounding lattice O atoms is established in the VO3′ configuration (see Figure S7). While the VO3′ configuration shows good stability, it also demonstrates an insufficient capacity for H_2_O adsorption, attributed to the coordination saturation of the Cu atom. The VO2′ configuration displays lattice rigidity in comparison to VO1′. Consequently, the VO2′ configuration lacks the structural flexibility exhibited by VO1′, making the H_2_O adsorption unstable.

In summary, since the active catalytic site needs to adsorb the H_2_O molecule stably, we choose the VO1′ configuration for the subsequent analysis.

### Dynamic Structure Induced by H_2_O Adsorption on the Cu-SAC

**Figure 3:**
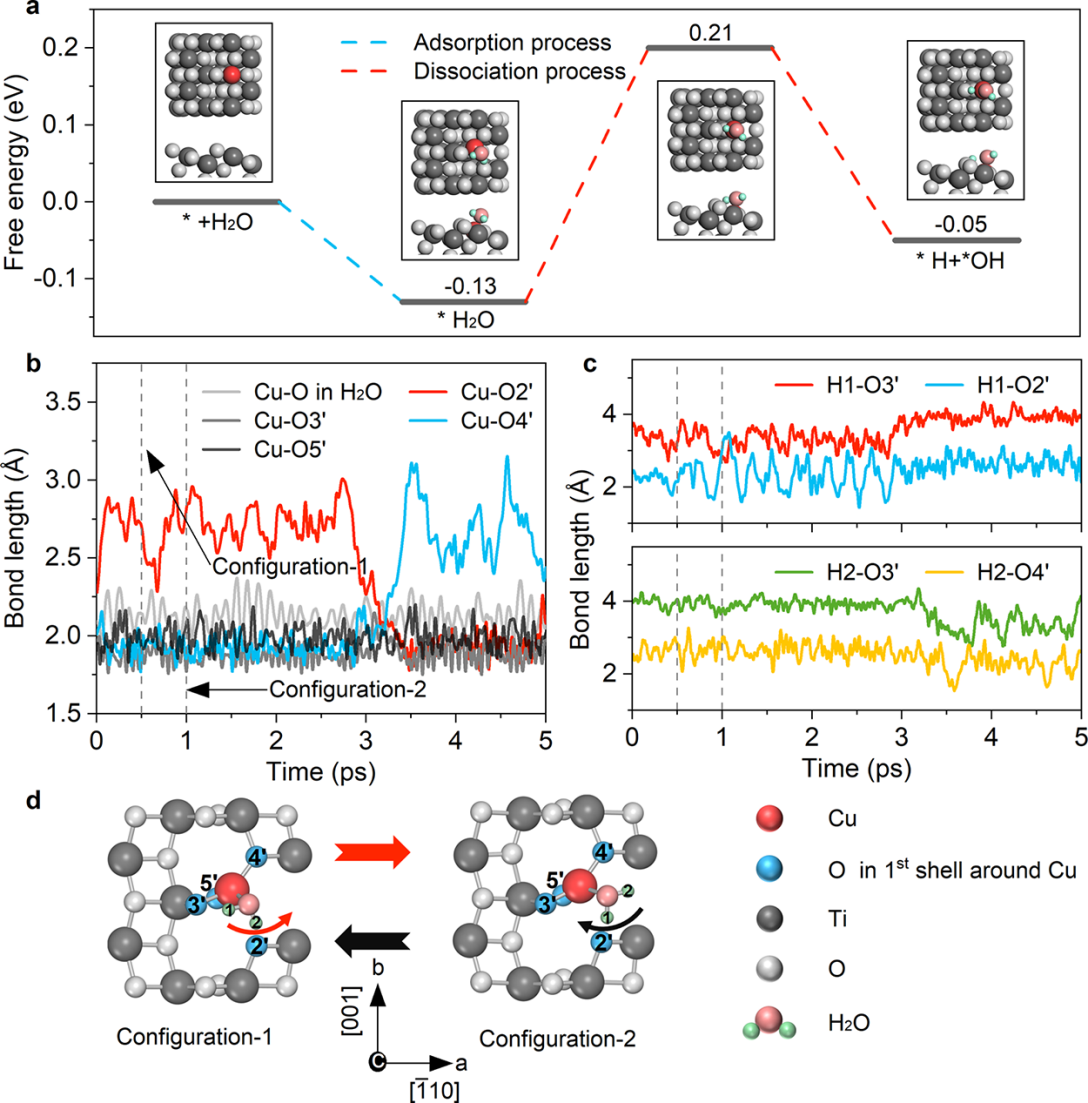
**Dynamic coordination structure of Cu-SAC after H**_**2**_**O adsorption.** (a) Free energy diagrams of the H_2_O adsorption and dissociation, including the molecular state, dissociative state, and transition state. (b, c) Evolution of the key bond lengths in canonical ab initio molecular dynamics simulation with the initial temperature of 300 K. Note that O2′ and O4′ are equivalent, and the switching of the Cu–O2′ and Cu–O4′ bond lengths at 3 ps creates a structure equivalent to that before 3 ps. (d) Two adsorption configurations, chosen prior to 3 ps in (b), and used as the initial structure for the H_2_O photodissociation dynamics shown in Figure 4e,f. The periodic transformation of the Cu–O coordination structure is illustrated by the changes in the bond length at 3 ps, as depicted in (b). (d) Statistical analysis of the H1–O3′ bond lengths during the 0–3 ps time frame reveals the existence of two predominant local structures preceding the periodic Cu–O structural changes. The statistical data for the H1–O3′ bond lengths is presented in Figure S9.

As depicted in Figure 3a, the transition state search suggests that the adsorbed H_2_O exists in a molecular state, wherein one H atom forms a hydrogen bond with the O2′ atom surrounding the Cu-SAC (refer to Figure 1c for the configuration). The energy barrier for H_2_O dissociation is 0.34 eV, which can be overcome through thermal catalysis. However, the energy barrier for the recombination of H and OH into H_2_O is smaller, suggesting a higher likelihood of the molecular form of H_2_O. Furthermore, a relatively small dissociation energy barrier indicates an intrinsic activity of the Cu-SAC structure with lattice O vacancies. However, to enhance the dissociation of H_2_O molecules, additional energy and charge must be provided through photocatalysis. In comparison, in the absence of an injected electron, thermal catalysis would promote H and OH recombination, as discussed further below.

To validate the dynamic adsorption configuration of the H_2_O molecule, we conducted a 5 ps microcanonical AIMD simulation. Figure 3b and 3c illustrate that within the initial 3 ps, the Cu atom is displaced from its original position due to hydrogen bond formation, creating a quasi-planar quadrilateral structure involving the four neighboring O atoms (O3′, O4′, O5′, and O in H_2_O). Over the subsequent 2 ps, the Cu–O4′ bond is replaced by the Cu–O2 bond. Given the structural equivalence of the O2′ and O4′ atoms, a comparable quasi-planar quadrilateral configuration emerges. This local structural transformation is further validated through a 30 ps AIMD simulation (refer to Figure S8).

To investigate the structure after H_2_O adsorption, we calculate the statistical distribution of the H1–O3′ bond length (Figure S9) during the initial 3 ps of the canonical AIMD simulation of Figure 3b. The analysis reveals two dominant adsorption configurations based on the length of the hydrogen bond between the H_2_O molecule and the Cu SAC surface. The two adsorption configurations are shown in Figure 3d. In conclusion, the AIMD simulation underscores a periodic reconstruction of local Cu–O coordination.

### Hot Electron Injection into the Hybridized Cu-H_2_O Orbital

**Figure 4:**
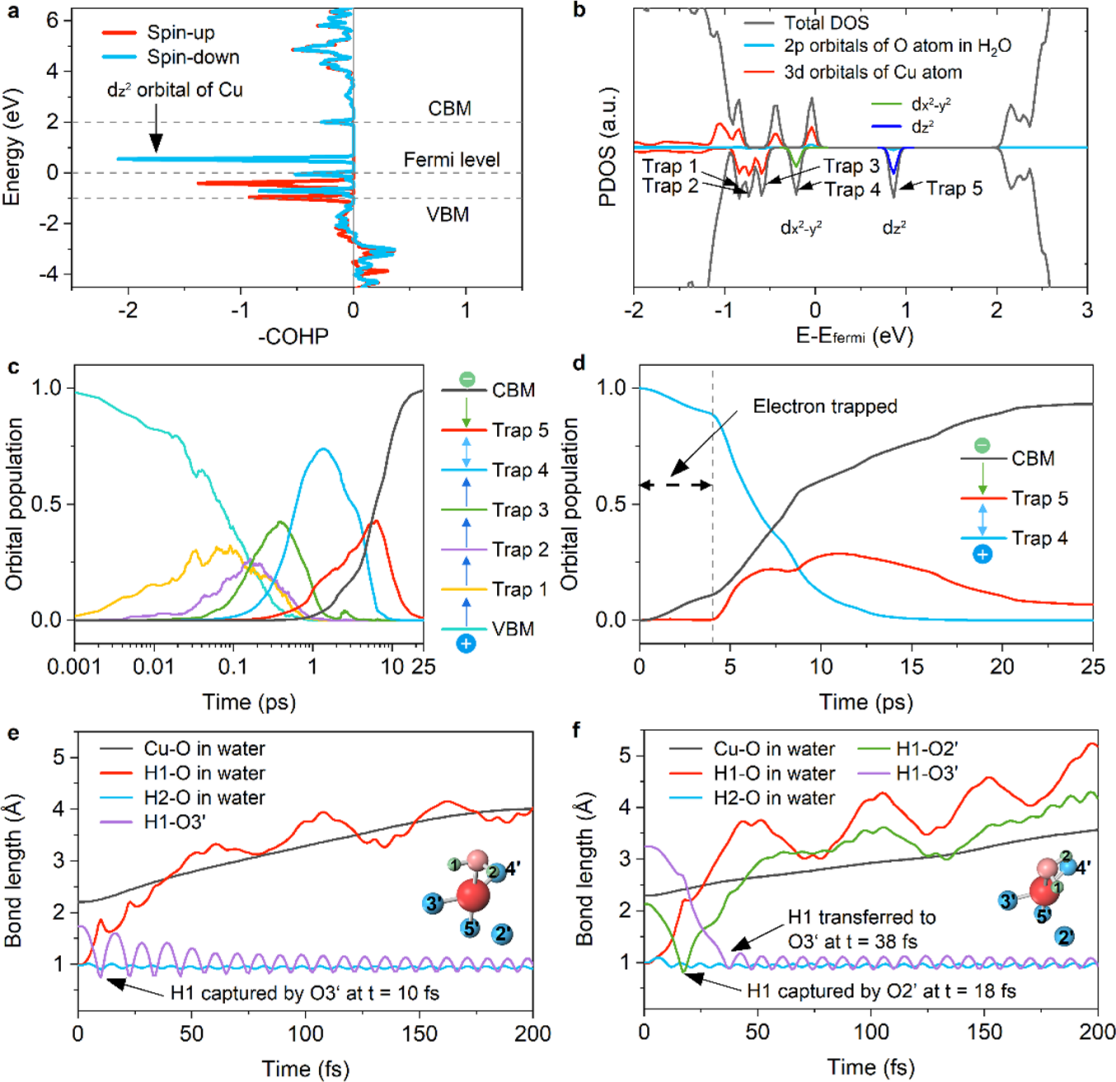
**Charge carrier dynamics and photoinduced H**_**2**_**O dissociation.** (a) Crystal orbital Hamilton population (COHP) between the Cu atom and the O atom of H_2_O. (b) Projected density of states (PDOS) of H_2_O adsorbed Cu-SAC/TiO_2_. (c) Hot electrons relax to the TiO_2_ CBM, hop to and accumulate in the Trap 5 state, and then recombine with holes that hop from the VBM to Traps 1–4, as illustrated in the inset. The time-dependent populations of different states provide insights into the dynamics of electron and hole trapping and recombination within the spin-down channel. (d) Starting with electrons in the CBM, the inset demonstrates electron trapping by Trap 5 and electron recombination with a hole in Trap 4. The time-dependent populations of various states elucidate the dynamics of electron and hole trapping and recombination, with emphasis on the transition involving Traps 4 and 5 and the CBM. (e, f) Following H_2_O adsorption and subsequent acquisition of hot electrons, H_2_O undergoes desorption from the catalyst surface and subsequent dissociation. The evolution of the Cu–O and H–O bond lengths after electron transfer to H_2_O is depicted for two representative initial configurations, as shown in Figure 3d.

Figure 4a illustrates the crystal orbital Hamilton population (COHP) between Cu and O in H_2_O^34−36^ after the adsorption of the H_2_O molecule onto the Cu atom. The corresponding PDOS is presented in Figure 4b. The results indicate that the hybridized orbital of H_2_O is in the spin-down channel and exhibits antibonding characteristic. The Cu atom-induced localized electronic states, labeled as Traps 1–5, span the bandgap from the VBM to the CBM. Thus, the hot electron in the CBM can transition to the midgap orbital hybridized between the Cu-SAC and H_2_O. Comparison of the data in Figure 4b and Figure S5c indicates that oxygen vacancies introduce additional electrons that occupy d_*x*^2^–*y*^2^_ orbitals. In Figure S10a, the time-dependent electron populations on various energy levels during the electron transition from the CBM back to the VBM are presented. Notably, the energy level corresponding to the highest empty hybridized orbital of H_2_O with Cu-SAC (Trap 5) can capture hot electrons, highlighting the ability of the Cu d_*z*^2^_ orbital to transfer a hot electron to the adsorbed H_2_O molecule.

Since the energies of the d orbitals of the Cu atom are lower than those of the Ti atoms, the orbitals formed by hybridization of the d orbitals of the Cu atoms and the H_2_O molecules form bandgap states, corresponding to the trap states in Figure 4b. The hybridization process is illustrated according to the ligand-field theory in Figure S1. After an electron is captured, the antibonding hybridized orbital, corresponding to the d_*z*^2^_ orbital closest to the CBM (Trap 5), can transfer the electron to the H_2_O molecule. This can be seen from the local charge distribution of Trap 5 shown in Figure S12. In addition, because the orbital is antibonding, the captured hot electron pushes the H_2_O molecule away from the surface, preventing the O atom formed after H_2_O dissociation from filling the original oxygen vacancy, thus avoiding deactivation of the single atom site.

The photogenerated holes accumulate at the VBM and can recombine with hot electrons in Trap 5 by transitions through Traps 1–4. We perform NAMD simulations to investigate this process. Figure 4c displays the time-dependent populations of the VBM, CBM and trap states. While the hole can efficiently transfer stepwise from the VBM to Trap 4 within approximately 1 ps, the transition to Trap 5 is significantly prolonged, taking around 10 ps, allowing buildup of the hot electron population in Trap 5. Thus, Trap 5 can effectively accumulate electrons over several picoseconds and shield them from recombination with holes, in agreement with the reported experimental results obtained by transient absorption spectroscopy.^22^ A closer examination of the electron trapping and recombination steps is shown in Figure 4d. The existence of a substantial hot electron population in the d_*z*^2^_ orbital of Cu hybridized with H_2_O over 15 ps creates the condition needed for electron transfer to H_2_O, initiating the photocatalytic reaction.

The non-adiabatic coupling (NAC) matrix elements between different energy levels in Figure S11 indicate a relatively higher transition probability between the VBM and Traps 1–4, while a smaller transition probability between Traps 1–4 and Trap 5. Additionally, as shown in Figure S12, the wavefunction of Trap 4 primarily distributes parallel to the catalyst surface, corresponding to the d_*x*^2^–*y*^2^_ orbital of Cu atoms, whereas the wavefunction of Trap 5 is predominantly oriented vertically to the surface, corresponding to the d_*z*^2^_ orbital of Cu atoms. Such arrangement, and in particular the distribution of the Trap 5 wavefunction, relies on the quasi-planar quadrilateral structure demonstrated in Figure 1c, underscoring the important role of the quasi-planar structure induced by the H_2_O adsorption in the photocatalytic hydrogen evolution on Cu-SAC/TiO_2_.

The electron-induced dissociation of H_2_O is simulated using the impulsive two-state (I2S) method (see Supporting Information and Figure S13 for details). Figure 4e and 4f depict H_2_O dissociation subsequent to the injection of an electron into the orbital hybridized between the Cu-SAC and H_2_O, starting from the two configurations shown in Figure 3d. In configuration 1, the H1 atom freed during the water dissociation is directly captured by the O3′ atom (bridging O atom) at *t* = 10 fs. In comparison, configuration 2 leads to capture of the H1 atom by the O2′ atom at *t* = 10 fs, followed by transfer to the O3′ atom at *t* = 38 fs. Application of the Hubbard U correction to the Cu atoms does not significantly alter the wavefunction distribution of the Trap 5 state (see Figure S14 for details), but rather elevates its energy level. Consequently, the energy gap between Trap 4 and Trap 5 is increased, slowing electron–hole recombination, while the energy of the trapped hot electron in the Trap 5 state grows, increasing the driving force of the photochemical reaction. Both factors facilitate a more efficient H_2_O dissociation process.

### Inert State of the Cu-SAC Induced by H Adsorption

The H_2_O dissociation results presented in Figure 4 indicate that the released H atoms can be captured by the lattice O atoms. An adsorbed H atom introduces an additional electron into the d_*z*^2^_ orbital of the Cu atom, as illustrated in Figure S15. The introduction of the extra electron prompts the Cu atom to repel the lattice O atoms (specifically O3′ and O5′) that lie within the d_*z*^2^_ orbital plane. Ultimately, this repulsion results in breaking of corresponding Cu–O bonds and the consequent transformation leading to the formation of a two-coordinated Cu atom displaying a linear configuration. This configuration aligns with the characteristics of Cu_2_O or Cu^+^, as indicated in the Mulliken charge analysis (Table S1). It is important to note that photogenerated electrons directly captured by the Cu atom can also induce the Cu atom to display the linear configuration. However, this process is expected to occur rapidly due to the ultrafast electron transition. In contrast, the experimental observations reveal a gradual enhancement of the Cu^+^ signal throughout the photocatalytic reaction. This phenomenon strongly suggests that the predominant cause of the Cu^+^ signal is the capture of H atoms by the lattice O atoms.

**Figure 5:**
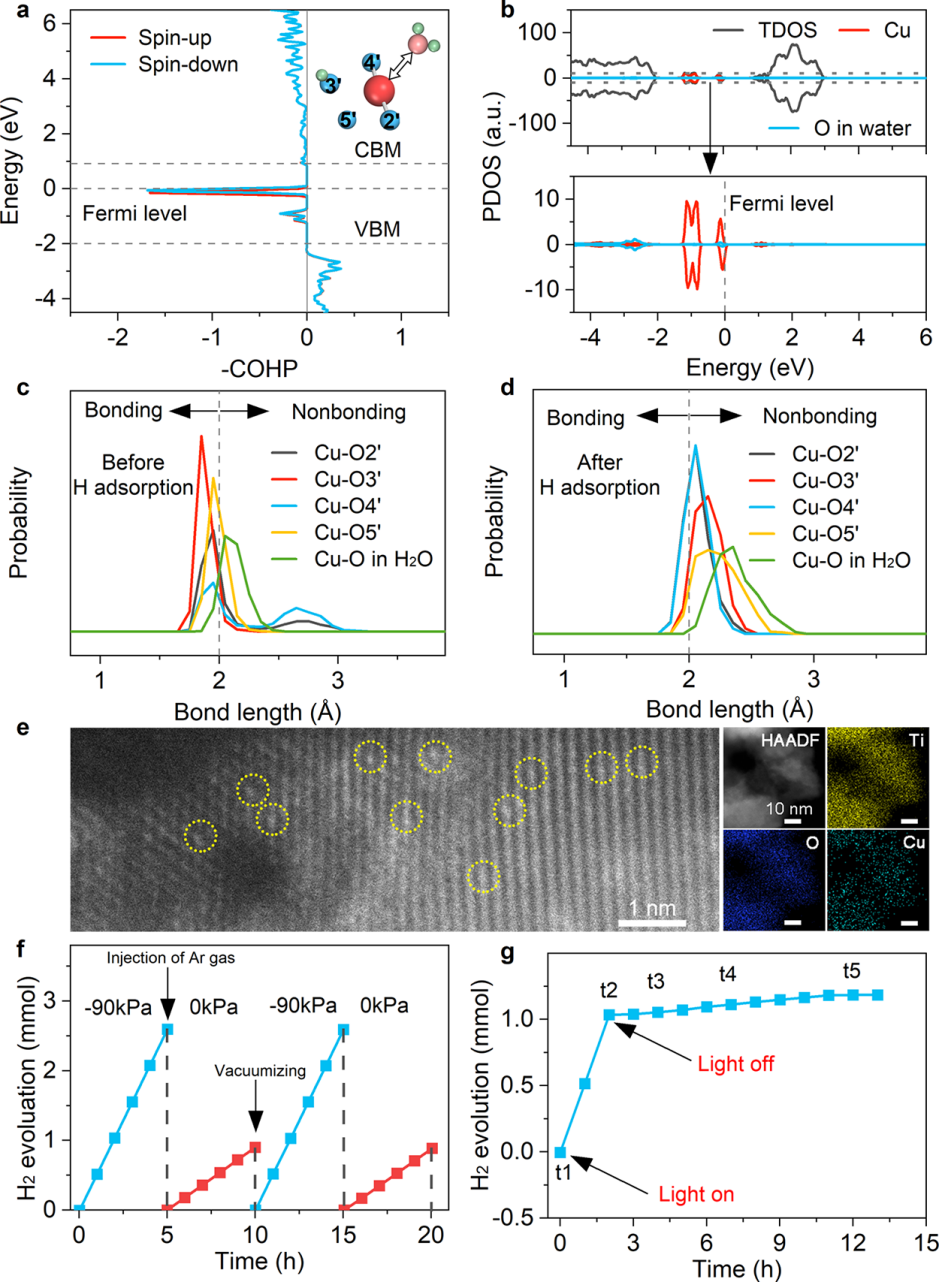
**Cu-SAC/TiO**_**2**_**passivation by H adsorption.** (a) Crystal orbital Hamilton population between the Cu atom and the O atom of H_2_O with an extra H atom adsorbed onto the neighboring bridging O atom of Cu-SAC. (b) PDOS of Cu-SAC/TiO_2_ with the adsorbed H atom. (c, d) Distributions of the Cu–O bond lengths before and after H adsorption at 300 K. The observed increase in the Cu–O bond lengths following the H adsorption suggests that the additional electron released from the H atom is injected into the d orbital of the Cu atom, thereby generating a repulsive force between the Cu atom and the H_2_O molecule. (e) High-resolution STEM and STEM-EDS mapping images of Cu-SAC/TiO_2_. (f) Photocatalytic H_2_ evolution experiments of Cu-SAC/TiO_2_ under dynamic pressures with Ar atmosphere protection. 0 kPa represents the standard atmospheric pressure. (g) H_2_ evolution with light on and off. The sensitivity of the photocatalytic performance of Cu-SAC to pressure suggests that the rate-determining step in photocatalytic hydrogen production involves H diffusion and desorption. Comparative data for Cu-SAC/TiO_2_ and TiO_2_ are presented in Figures S22 and S23.

The simulated absorption spectra of Cu-SAC/TiO_2_ before and after the H adsorption (Figure S16) clarify the origin of the color alteration during the reaction process. Namely, H adsorption is the reason the Cu-SAC turns black. An additional, crucial aspect arises at this stage: the Cu-SAC becomes passivated because of the repulsive force to adsorption of a H_2_O molecule (Figure 5a,b). The COHP analysis, displayed in Figure 5a and detailed in Table S2, reveals a significant reduction in the strength of the chemical bond between the Cu atom and the adjacent H_2_O molecule as a result of adsorption of the extra H atom. A comparison of the 3 ps microcanonical AIMD simulations conducted before and after the adsorption of the H atom underscores that H adsorption leads the system to maintain a relatively large distance between the H_2_O molecule and the catalyst surface. This is demonstrated in Figure 5c,d, as well as in Figures S17 and S18. The analysis underscores that the H atom adsorbed onto the neighboring bridge O atom prompts a transformation of the Cu atom into an inert state. Only after the H atom desorbs from the catalyst surface can a new reaction cycle be initiated.

### Experimental Verification

Given that the desorption of hydrogen from the TiO_2_ surface necessitates surpassing an energy barrier of at least 1 eV,^37^ it is reasonable to infer that the hydrogen desorption may serve as the rate-determining step in the process of hydrogen production on the Cu-SAC. If this is indeed the case, altering the conditions of the photocatalytic process, such as lowering the pressure, can enhance the reaction rate.

To experimentally validate the analysis, we synthesize a highly active Cu-SAC/TiO_2_ composite (Figures S19 and S20). The homogeneous incorporation of individual Cu atoms within Ti vacancies is confirmed through atomic-resolution high-angle annular dark-field (HAADF) STEM imaging, as illustrated in Figure 5e. Subsequently, a series of controlled experiments aimed at the photocatalytic hydrogen evolution are conducted. Initially, the gradual conversion of Cu^2+^ to Cu^+^ during the reaction is confirmed using in situ electron paramagnetic resonance (EPR) (Figure S21). We then proceeded to explore the impact of pressure. As depicted in Figure S22, an increase in pressure corresponds to a rapid decline in the photocatalytic activity of Cu-SAC/TiO_2_, while the pure TiO_2_ catalyst exhibits negligible variations under various pressures, as shown in Figure S23. This observation underscores the adverse influence of slow H atom desorption on hydrogen evolution over Cu-SAC/TiO_2_ under high pressure. The experiment involving dynamic pressure changes, as presented in Figure 5f, also reaffirms this effect.

Even after illumination stops, the production of the H_2_ gas (depicted in Figure 5g) continues at a slow rate and eventually stops after several hours. Simultaneously, the initially black catalyst reverts to its original white color (Figure S24). These phenomena collectively indicate the formation of passivated Cu atoms during the photocatalytic reaction, emphasizing the pivotal role of H desorption from neighboring O atoms in maintaining reaction continuity. Hence, it is imperative to enhance the desorption rate of H atoms, a factor that can be modulated by altering the system pressure or temperature.^38,39^

One of the major difficulties in the application of SACs is their insufficient lifetime and stability. For instance, in SACs supported on C_3_N_4_, the M–C or M–N bonds are easily affected by heat or photoelectric effects. The energies of the M–C and M–N bonds are relatively low, and it is easy for isolated atoms to migrate and form clusters, leading to deactivation. In comparison, single atoms are anchored on metal oxide substrates by M–O bonds, which have high energy and strongly resist deactivation. In addition, Cu initially exists in a four-coordinate state in the Cu-SAC studied here. However, after one reaction cycle, the H adsorption induces the Cu atom to enter the two-coordinate state. Although the coordination number decreases, a protective electronic layer is formed around Cu, preventing further contact with H_2_O and capture of more charge, thus avoiding a further decrease in the coordination number. Therefore, single atoms supported on metal oxides have good stability. In our experiments, no significant deactivation was observed after 20 h of changing pressure and irradiation conditions (Figure 5f).

## Discussion

The comprehensive process of photocatalytic hydrogen evolution through water splitting on Cu-SAC is outlined in Figure 1. Our findings demonstrate that the flexible interaction between Cu-SAC and lattice O atoms establishes an active site for H_2_O molecule adsorption. Facilitated by a hydrogen bond between H_2_O and lattice O atoms, this interaction results in the formation of a quasi-planar structure, creating a hybridized Cu/H_2_O orbital below the TiO_2_ CBM. The photogenerated hot electrons captured by the Cu d_*z*^2^_ orbital can be injected efficiently into the H_2_O molecule. Additionally, H atoms released during the H_2_O dissociation can be adsorbed onto lattice O atoms, inducing a transformation of the coordination configuration of the Cu atom from CuO to Cu_2_O. These findings align with the XANES^21^ and EPR^22^ characterizations. In contrast to the proposed beneficial role of Cu^+^,^21,22^ we illustrate that Cu^+^ is an inert state of the Cu-SAC appearing during the reaction. To enhance the efficiency of the photocatalytic hydrogen evolution, it becomes essential to accelerate the rate of desorption of H atoms, a task achievable by reducing system pressure or elevating reaction temperature.^38,39^

Regarding transition metal cocatalysts present in the bulk phase: small molecules can interact with a cluster of metal surface atoms. In contrast, when a cocatalyst comprises only a single atom, the interaction of small molecules is confined to a subset of the d orbitals,^15^ as some of the d orbitals are already engaged in bonding with the substrate material.^40−42^ Accounting for the energy splitting of d orbitals through the ligand-field theory, the frontier orbitals of a metal SAC loaded onto TiO_2_ or other metal oxides with an octahedral structure include d_*x*^2^–*y*^2^_ and d_*z*^2^_ orbitals. NAMD calculations for Cu-SAC/TiO_2_ reveal that the hot electron is trapped by the d_*z*^2^_ orbital of the transition metal atom, thus creating an efficient bridge for charge transfer to the adsorbed molecule.^12^ Accordingly, we propose the following formulation to estimate the activity of SAC loaded on an octahedral metal oxide: a subset of d orbitals of a single transition metal atom ≈ an electron trap state ≈ a charge transfer bridge. Cu-SAC/TiO_2_ is the case in point. If the Cu atom substitutes Ti_5c_, the Cu d_*z*^2^_ energy level becomes excessively low to effectively capture high-energy hot electrons. Consequently, the trapped hot electrons lack the energy necessary to initiate H_2_O dissociation. However, the presence of a neighboring oxygen vacancy results in the release of two extra electrons, which occupy the d_*x*^2^–*y*^2^_ orbital, regardless of whether the d_*x*^2^–*y*^2^_ orbital is initially higher or lower in energy than the d_*z*^2^_ orbital. The preferred position of the d_*z*^2^_ orbital closer to the CBM of the substrate material endows Cu-SAC/TiO_2_ with an excellent photocatalytic activity. The notion of the d_*z*^2^_ orbital of SAC/TiO_2_ functioning as a descriptor for the hot electron trap state driving the photochemistry can be extended to other SACs involving different substrate catalysts.

It is important to recognize the synergistic effect of the quasi-planar Cu–O coordination structure and the d_*z*^2^_ orbital as electron transport bridge. Considering the potential of the d_*z*^2^_ orbital to capture electrons, it is natural to expect that H_2_O can be adsorbed onto Cu atoms through the d_*z*^2^_ orbital to form a hybridized molecular orbital, thereby effectively utilizing the charge captured by d_*z*^2^_. However, because the d_*z*^2^_ orbital has an antibonding property, five-coordinated Cu atoms cannot facilitate effective adsorption. At the same time, when there is an oxygen vacancy around the Cu atom, the four-coordinated Cu–O structure gains sufficient flexibility. Although the d_*z*^2^_ orbital still has an antibonding property, H_2_O can stably form a quasi-planar structure with the help of hydrogen bonding and adsorb onto the Cu atom through the d_*z*^2^_ orbital. Considering that SACs supported on metal oxide substrates typically exhibit an octahedral metal–oxygen structure, SACs that replace cations of metal oxides are often unable to generate sufficient adsorption capacity in the absence of oxygen vacancies. Therefore, even if they can capture charges, the charge cannot be effectively used to drive reactions. The effects of the local structure of “oxygen vacancies + single atoms” on adsorption and charge capture studied in this paper can be extended to other similar catalysts.

## Conclusion

We investigated the reversible dynamic coordination changes in Cu-SAC/TiO_2_ and their correlation with different steps of the photocatalytic hydrogen evolution mechanism. The reported findings underscore the pivotal role of the local Cu–O coordination structure in the heightened photocatalytic activity of Cu-SAC/TiO_2_. The simulations show that the Cu d_*z*^2^_ orbital effectively captures photogenerated electrons and injects them into the H_2_O molecule, promoting the water-splitting reaction.

The H atom generated during the Volmer process adsorbs to a bridging O atom neighboring the Cu-SAC, resulting in changes in the valence state and color of Cu. The H atom adsorption does not activate Cu-SAC/TiO_2_. Conversely, it induces an inert state in Cu by introducing an additional electron into the Cu d_*z*^2^_ orbital. Consequently, the corresponding color and valence state changes serve as indicators of the photocatalytic process rather than the origin of hydrogen evolution. The study provides a novel perspective on the mechanism of water splitting on SACs and offers valuable guidelines for the design of SACs for photocatalytic reactions.

## Supporting Information Available

Description of the simulation methodologies and Figures S1–S24 and Tables S1 and S2 providing additional theoretical and experimental results (PDF)
