# Supplementary material for: dz2 Band Links Frontier Orbitals and Charge Carrier Dynamics of Single-Atom Cocatalyst-Aided Photocatalytic H2 Production
Source: J Am Chem Soc. 2023 Dec 12;145(51):28166–75. doi: 10.1021/jacs.3c10661 (PMC10755699; doi:10.1021/jacs.3c10661)
Supplement: Supplementary file 1 — ja3c10661_si_001.pdf [file ja3c10661_si_001.pdf]

Supporting Information for

d_{z^2} Band Links Frontier Orbitals and Charge Carrier Dynamics of Single-Atom Cocatalyst-Aided Photocatalytic H_2 Production

Yiwei Fu,[a,¶] Kejian Lu,[a,¶] Anlan Hu,[a] Jie Huang,[a] Liejin Guo,[a] Jian Zhou,[b] Jin Zhao,[c,d] Oleg V. Prezhdo,[e,*] Maochang Liu[a,f,*]

[a]*International Research Center for Renewable Energy, State Key Laboratory of Multiphase Flow, Xi'an Jiaotong University, Xi'an, Shaanxi 710049, P. R. China*

[b]*Center for Alloy Innovation and Design, State Key Laboratory for Mechanical Behavior of Materials, School of Materials Science and Engineering, Xi'an Jiaotong University, Xi'an, Shaanxi 710049, China*

[c]*ICQD/Hefei National Laboratory for Physical Sciences at the Microscale, and CAS Key Laboratory of Strongly-Coupled Quantum Matter Physics, and Department of Physics, University of Science and Technology of China, Hefei, Anhui 230026, China*

[d]*Synergetic Innovation Center of Quantum Information & Quantum Physics, University of Science and Technology of China, Hefei, Anhui 230026, China*

[e]*Departments of Chemistry, and Physics and Astronomy, University of Southern California, Los Angeles, CA 90089, USA*

[f]*Suzhou Academy of Xi'an Jiaotong University, Suzhou, Jiangsu 215123, P. R. China*

[¶]These authors contributed equally.

[*]To whom correspondence should be addressed.

E-mail: prezhdo@usc.edu (Oleg V. Prezhdo), maochangliu@mail.xjtu.edu.cn (Maochang Liu)

Calculation Methods

Details of DFT calculations

The ab initio NAMD study uses density functional theory (DFT) as implemented in the Vienna Ab initio simulation package (VASP) to carry out the static and ab initio molecular dynamics (AIMD) calculations[1-4]. The spin-unrestricted DFT calculations employ the projector augmented wave (PAW) method[5, 6] and the Perdew-Burke-Ernzerhof (PBE) exchange-correlation functional[7], and account for van der Waals (vdW) interactions using DFT-D3[8]. The energy cutoff for the plane wave basis was set to 520 eV. A 2 × 2 supercell containing 72 atoms is employed to model the anatase $TiO_2(101)$ surface, with a 15 Å vacuum layer added between periodic slabs. A Γ-centered 3 × 3 × 1 Monkhorst−Pack k-point mesh was used to optimize geometry; the electronic structure of the system is obtained by sampling the Brillouin zone Γ-centered 9 × 9 × 1 k-point mesh. The bottom layer Ti and O dangling bonds are saturated with pseudo-hydrogens with nuclear charges of +1.33e and +0.66e, similar to the protocol of Kowalski and co-workers[9]. Due to the self-interaction error in DFT, the electronic bandgap is usually underestimated in metal oxides. To correct the self-interaction error of the DFT functional, we use the DFT + U method to treat the d electrons of Ti atoms (U = 4.5 eV) [10, 11]. As to the single metal single atoms working as catalysts, previous studies on TiO_2 indicate that alignment of the relevant energy levels at the molecule/TiO_2 interface are affected much less[12, 13]. To confirm this point, we have compared the density of states of the Pt single atom and H_2O adsorbed on the Pt atom using DFT and DFT + U (U=4.5 eV) and have observed only minor differences (Supplementary Fig. S1). We have also tested the importance of the U correction for the d electrons of the Cu atom (U = 5.2 eV) [11] (Supplementary Fig. S14). The energy gaps between the TiO_2 CBM and the $d_{x^2-y^2}$ and d_{z^2} states of Cu undergo minor changes upon inclusion of the U correction for Cu. The trap states formed by the Cu $d_{x^2-y^2}$/ d_{z^2} orbitals hybridized with the H_2O orbitals both with and without the Cu U correction. The key steps of the photoinduced dissociation of the water molecule involve photogeneration of an electron in the TiO_2 CBM, electronic transition from the CBM to the Cu(d_{z^2}) trap state, and subsequent electron transfer into the H_2O molecule, initiating the H_2O dissociation. Because the energy gaps and charge densities of these key states are similar with and without the U correction on the Cu atom, the conclusions obtained in the manuscript should be independent of the correction.

Free energy calculation

The free energy for adsorption and reaction is defined as

$$\Delta G = E_{substrate+\mathrm{adsorbate}} - E_{substrate} - E_{\mathrm{adsorbate}} + \Delta E_{ZPE} + T\Delta S \quad \text{(S1)}$$

where $E_{substrate+\mathrm{adsorbate}}$, $E_{substrate}$ and $E_{\mathrm{adsorbate}}$ are the energies of catalyst with adsorbate, pristine catalyst and adsorbate, respectively. ΔE_{ZPE} and ΔS are the difference in zero-point energy and adsorbate entropy between the adsorbed and the reactant structures. T is temperature.

Molecular dynamics

After the geometry optimization, we use velocity rescaling to bring the system temperature to 300 K. Then, we then conduct constant temperature AIMD to observe the dynamic change in the structure caused by H_2O adsorption. A time step of 1 fs was used for all the AIMD[1-4].

NAMD simulation

To investigate the excited state dynamics, which govern the lifetime of hot electrons and dissociation of H_2O, we use the ab initio NAMD simulations with the Hefei-NAMD code within the time-dependent Kohn-Sham (TDKS) framework[14]. In this mixed quantum-classical method, nuclei are treated classically based on the AIMD trajectory and the electron evolution is calculated within the quantum framework using the TDKS equation and surface hopping [15, 16]. Thus, the electron-phonon coupling can be considered in a time and configuration dependent manner. A 20 ps microcanonical AIMD trajectory is generated using a time step of 1 fs, and then, the NAMD results are obtained by averaging over 100 different initial configurations selected from the trajectory. For each chosen structure, we sample 2×10^4 realization of the surface hopping algorithm based on the classical path approximation.

In the ab initio NAMD simulation, the evolution of the excited electron or hole is based on the TDKS equation[13]:

$$i\hbar \frac{\partial \Phi_p(r,t)}{\partial t} = H(r, R(t))\Phi_p(r,t) \quad \text{(S2)}$$

where $\Phi_p(r,t)$ is the wavefunction of the electron and R are atomic positions. $\Phi_p(r,t)$ can be expanded in the basis of time-independent Kohn-Sham orbitals, φ_j:

$$\Phi_p(r,t) = \sum_j c_j(t)\varphi_j(r; R(t)) \quad \text{(S3)}$$

By inserting expansion S3 into equation S2, one obtains the following equation for the expansion coefficients:

$$\dot{c}_j(t) = \frac{c_j(t) E_j(R(t))}{i\hbar} - \sum_i c_j(t) d_{ij}(R(t)) \tag{S4}$$

where $d_{ij} = \left\langle \varphi_i \left| \frac{\partial}{\partial t} \right| \varphi_j \right\rangle$ represents the nonadiabatic coupling (NAC), which governs nonradiative transitions between electronic states.

According to the FSSH algorithm[14], the probability of hopping between states *i* and *j* within a time interval *dt* is

$$g_{ij} = \frac{-dt \cdot b_{ji}(t)}{\left|c_j(t)\right|^2} \tag{S5}$$

where $b_{ji}(t) = -2\,\mathrm{Re}\left(c_j^*(t) c_i(t) d_{ij}(R(t))\right)$.

Impulsive two-state (I2S) model

In order to describe H_2O dissociation induced by injection of a hot electron we use the impulsive two-state (I2S) model. The injection of a hot electron is modelled by the anionic pseudopotential method[13], in which one 1s electron is excited from the core part and is placed in the valence electron orbital of H_2O by a pseudopotential method. The I2S model for simulating photochemical reactions has been successful in the previous works on chemical dynamics[17-19].

The idea of the I2S model is to simulate the electron-induced molecular reaction based on simple AIMD simulations. Illustrated in Supplementary Fig. 13a, the I2S approach involves four steps[18]. The system is excited from the ground state potential energy surface (PES) to the anionic potential energy surface (PES*). This is achieved by promoting one of the molecule core electrons to a valence electron orbital. After the evolution of time t*, the system goes back to the ground PES, where the energy and momentum gained from the excited state evolution can promote the molecule to overcome the reaction barrier. Here, we simulate H_2O^- by exciting a 1s electron from the core and to the H_2O valence electron orbital, such that the electronic configuration of the O atom in H_2O changes from $O1s^2 2s^2 2p^4$ to $O1s^1 2s^2 2p^5$.

The method we use in this work is modified from the original I2S model. Since in the Volmer step of water splitting ($H_2O+e^- \rightarrow OH^- + H$), the H_2O molecule captures and keeps the electron, there is no need to revert to the ground PES after H_2O is exited to the PES*. The I2S method used in this work is illustrated in Supplementary Fig. 13b[17, 19].

Experiment Methods

Materials preparation

All reagents were analytical grade and used without further purification. Tetraisopropyl titanate Ti $(OC_4H_9)_4$, methanol, terephthalic acid (H_2BDC), N,N-dimethylformamide (DMF) and $Cucl_2\cdot3H_2O$ were all purchased from Shanghai Sinopharm Chemical Reagent Co., Ltd. and the Ar gas (99%) was obtained from the Fast Gas Co., LTD. Xi 'an, Shaanxi. The water used in the experiments is deionized water with a resistivity of 18.2 MΩ cm.

MIL-125 (Ti) MOF synthesis

The MIL-125 (Ti) MOF was prepared by the method reported by our group with some modifications[20]. 3g of H_2BDC were introduced into a solution containing 54 mL of DMF and 6 mL of CH_3OH. The mixture was pretreated by magnetic stirring at 25 °C for 30 min, resulting in a transparent homogeneous solution. The 1.0 mL Ti $(OC_4H_9)_4$ was then added into the above mixture under the stirring condition for another 30 min. The solution was subsequently transferred to a 100 mL Teflon-lined stainless-steel autoclave which was placed in a 150 °C furnace. The reaction was allowed for 16 h and then cooled to room temperature naturally. The obtained product was thoroughly washed by DMF and methanol, and then went through centrifugation 3 times. The obtained solid precipitate was dried in the vacuum oven at 80 °C for 10 hours. The final obtained sample was MIL-125 (Ti) MOF.

Cu-SAC/TiO_2 synthesis

Cu-SAC/TiO_2 was prepared by the two-step method of Coordination-Cleavage with some modifications, as reported[21]. 100 mg MIL-125 (Ti) powders were first dispersed thoroughly into 100 mL of water with magnetic stirring, and a $CuCl_2\cdot3H_2O$ aqueous solution (10mg/mL, 0.75wt% Cu) was dropped into the above mixture. Then, the mixture was stirred for 4 more hours under the same conditions, to allow Cu^{2+} to be fully adsorbed to the Ti vacancy sites on the surface of the MOF. Finally, Cu-SAC/MIL-125 (Ti) was obtained by centrifuging and drying in the vacuum oven at 60 °C for 10 hours. Cu-SAC/TiO_2 was then prepared by placing Cu-SAC/MIL-125 (Ti) in a muffle furnace under high temperature annealing at 450 °C for 4 hours with the heating rate at 2 °C /min. The final photocatalyst was obtained for further use.

Photocatalytic test

The photocatalyst activity test was carried out on a gas-closed automatic sampling, negative pressure test system with a top irradiation Pyrex cell. The light source was the 300 W Xe-300BF

Xenon lamp from the Perfect Light. Before the irradiation, 20 mg Cu-SAC/TiO_2 catalyst was dissolved into 100 mL methanol (67 vt%) and deionized water solution. Upon ultrasonic treatment for 15 minutes, this homogeneous solution was transferred to the quartz glass reactor. After being evacuated by the vacuum pump, the pressure of the total system reached -90kPa (0kPa represents the standard atmosphere pressure) and remained unchanged for more than half an hour. The photocatalysts were irradiated by the Xe-lamp. Meanwhile, automatic sampling and timing were started. The pressure adjustment within the system was achieved by controlling the Ar gas flow rate under the condition of the vacuum pump pumping air all the time, preventing air from entering the system during the whole testing process. The reaction temperature was controlled by the ongoing cooling water system[22]. All the sample tests took place under the same conditions and operation.

Materials Characterization

X-ray diffraction (XRD) measurements were carried out by using the PANalytical X'pert MPD Pro diffractometer, with Cu Kα irradiation (λ = 1.5406 Å). The crystallite morphology was determined by using the JEOL 7800F scanning electron microscope (SEM) and the FEI Tecnai G2 F30 S-Twin transmission electron microscope (TEM). The EPR test was recorded on the Brucker EPR A200 spectrometer with the largest magnetic field intensity reaching 14500G.

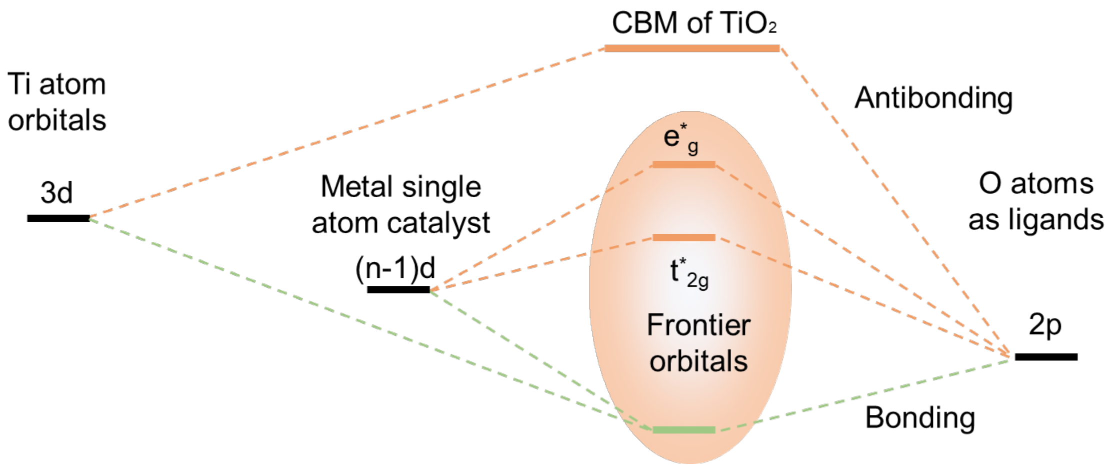

FIG. S1. Ligand-field theory illustration of the bonding orbitals for different single metal atoms substituting Ti_{5c} and forming an octahedral complex $[MO_5(H_2O)]$.

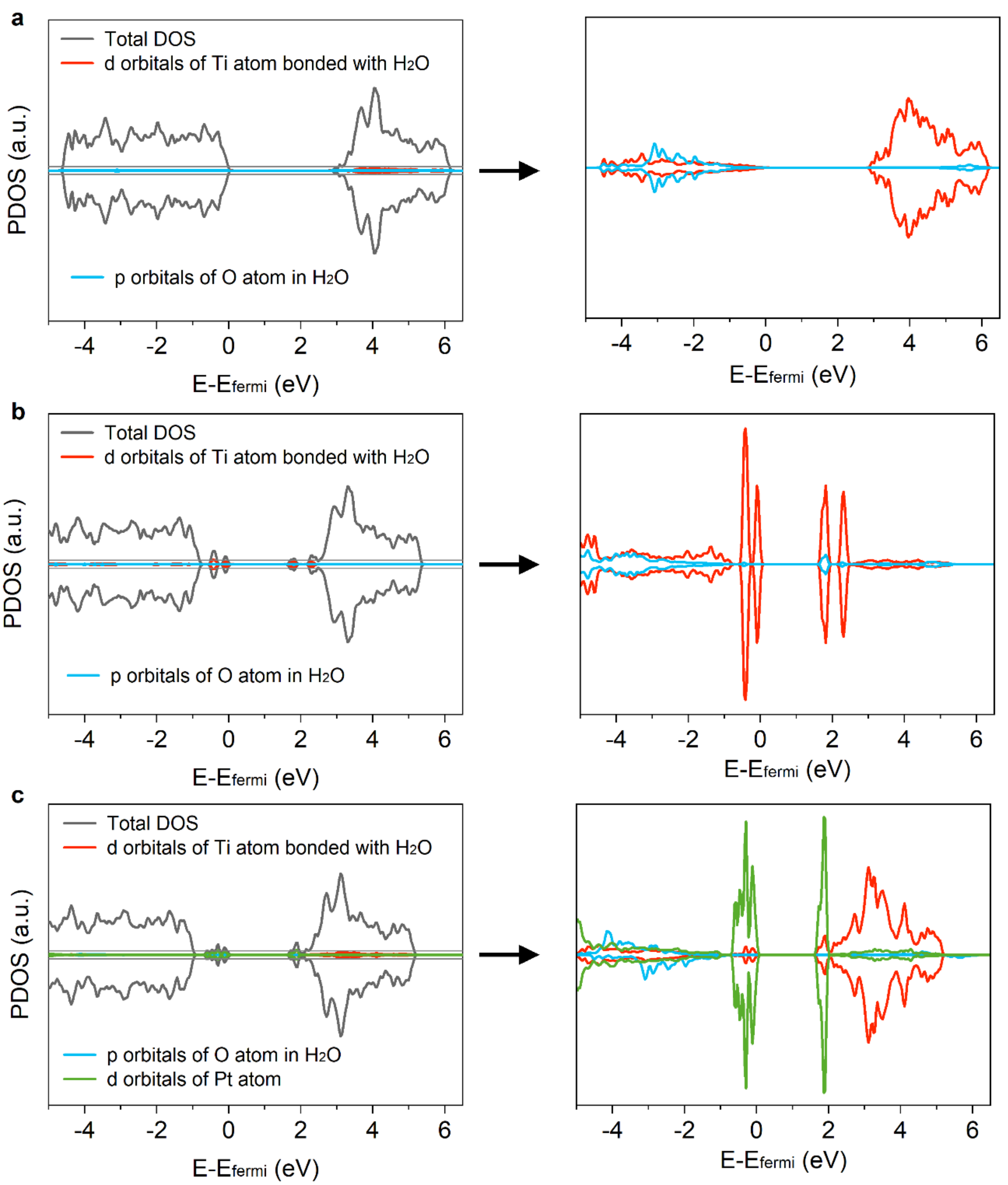

FIG. S2. PDOS of pure TiO_2 **(a)** and Pt-SACs/TiO_2 **(b, c)** with adsorbed H_2O. **(a)** H_2O adsorbed on Ti_{5c}. **(b)** H_2O adsorbed on Pt-Ti_{5c} (Pt substituting Ti_{5c}). **(c)** H_2O adsorbed on the Ti_{5c} next to the Pt single atom (substituting Ti_{6c}).

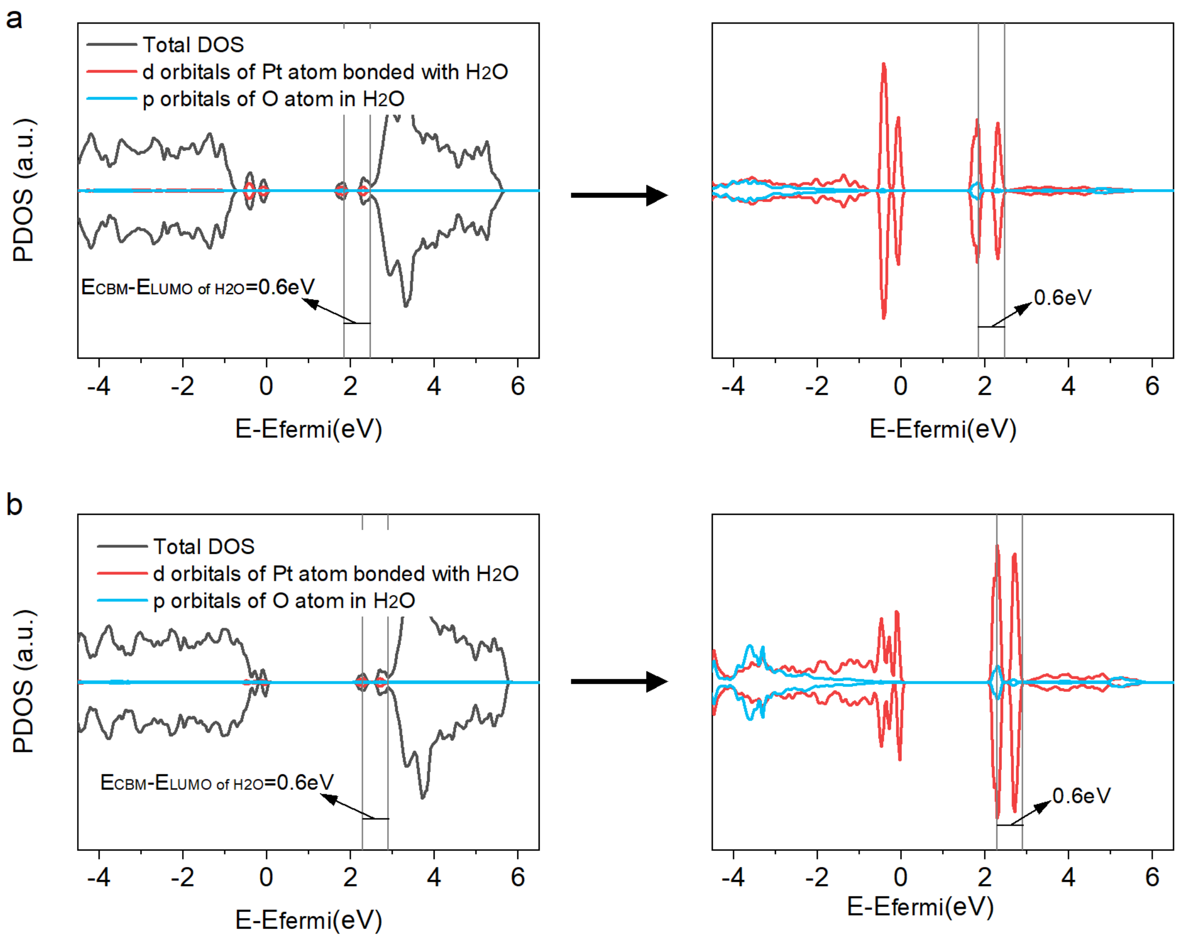

FIG. S3. PDOS of the TiO_2 anatase (101) surface with a single Pt atom substituting Ti_{5c} and adsorbed H_2O obtained using DFT **(a)** and DFT+U **(b)**. The zero energy is put at the Fermi level.

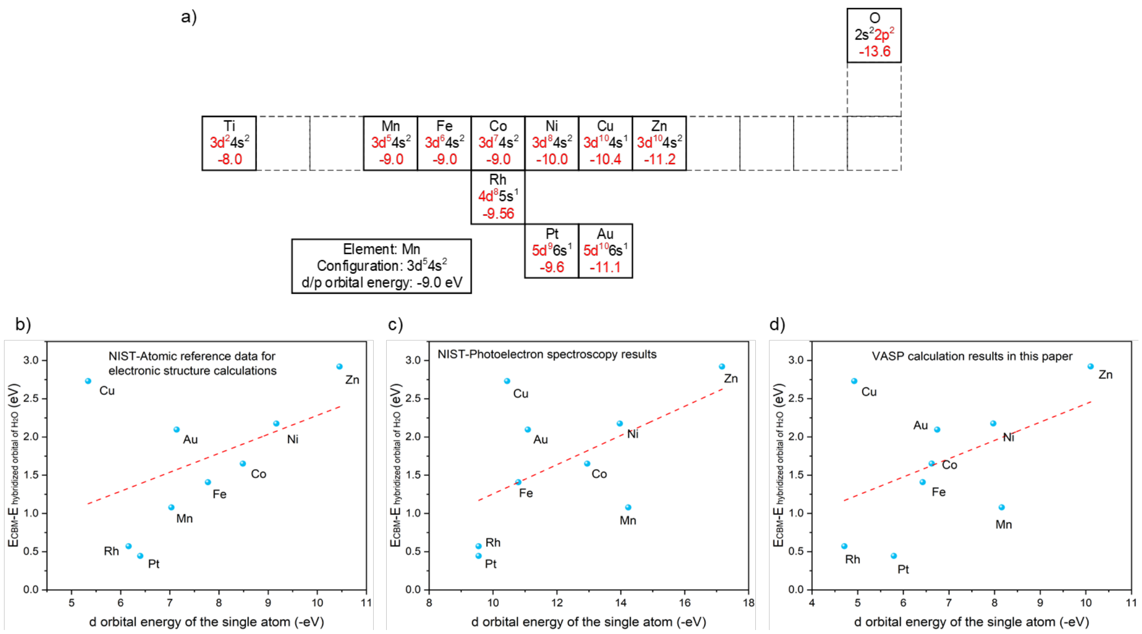

FIG. S4. **(a)** d orbital energies for different neutral metal atoms and p orbital energy for the O atom. The data is from ref [28] in the manuscript. **(b-c)** Positive correlation between the energy of the hybridized orbitals of H_2O with SAC and the d orbital energy from different sources. The d orbital energy results are from **(b)** NIST Atomic Spectra Database. Available: https://physics.nist.gov/asd. **(c)** NIST Atomic Reference Data for Electronic Structure Calculations. Available: https://www.nist.gov/pml/atomic-reference-data-electronic-structure-calculations. **(d)** Calculation results from this paper.

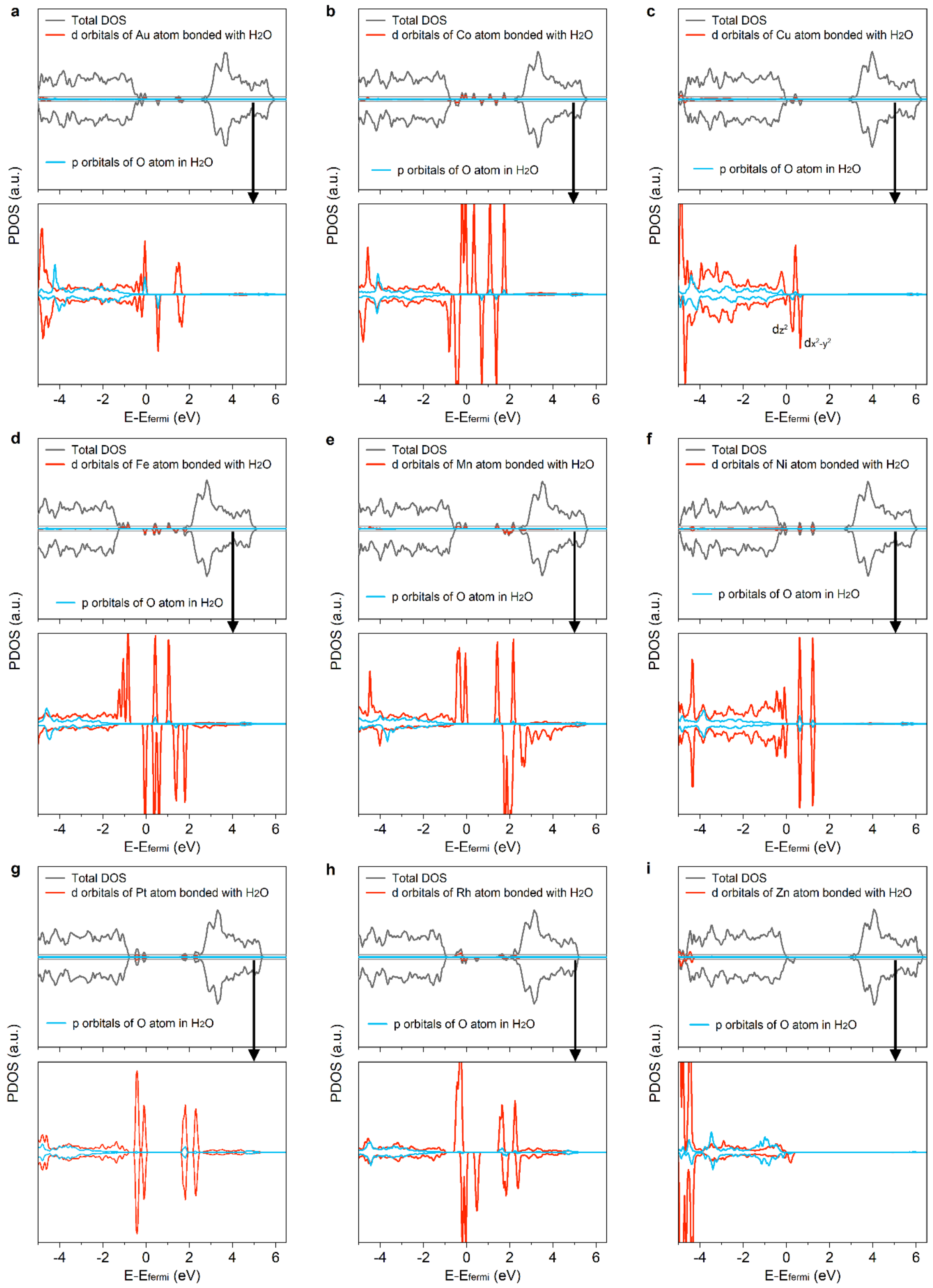

FIG. S5. PDOS for different metal-SACs on TiO_2. **(a)** Au. **(b)** Co. **(c)** Cu. **(d)** Fe. **(e)** Mn. **(f)** Ni. **(g)** Pt. **(h)** Rh. **(i)** Zn.

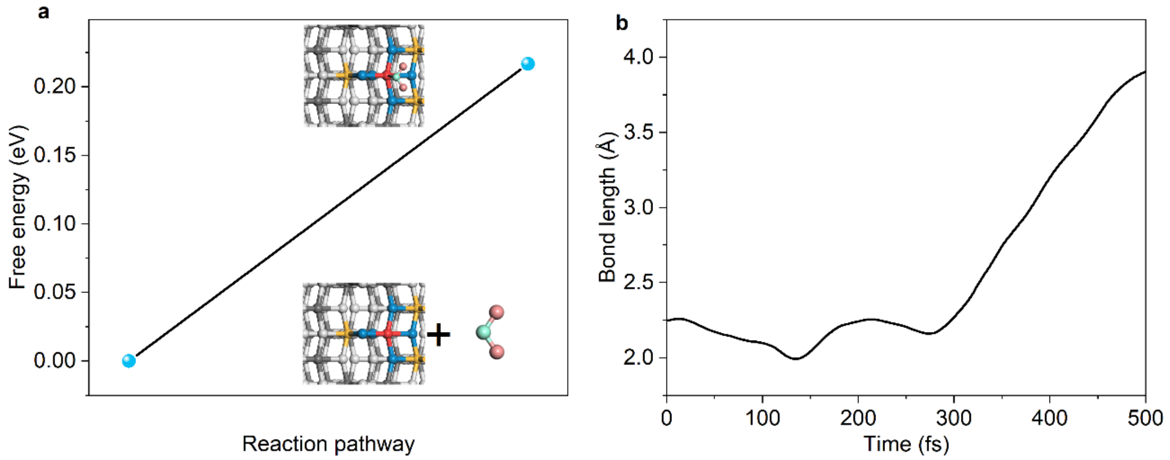

FIG. S6. H_2O adsorption on Cu-Ti$_{5c}$ (Cu substituting Ti$_{5c}$). **(a)** Free energy difference before and after H_2O adsorption onto Cu-Ti$_{5c}$. **(b)** H_2O molecule desorbing from Cu-Ti$_{5c}$ in canonical AIMD with the initial temperature of 300 K.

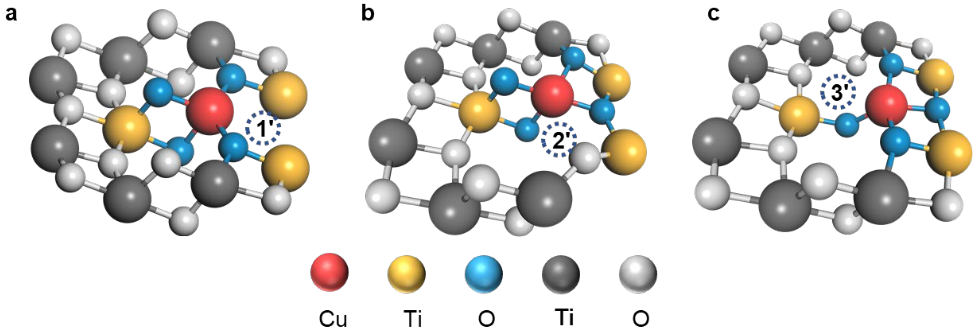

FIG. S7. Different configurations for the O atom vacancy (marked by the dashed circle). **(a)** VO1'. **(b)** VO2'. **(c)** VO3'.

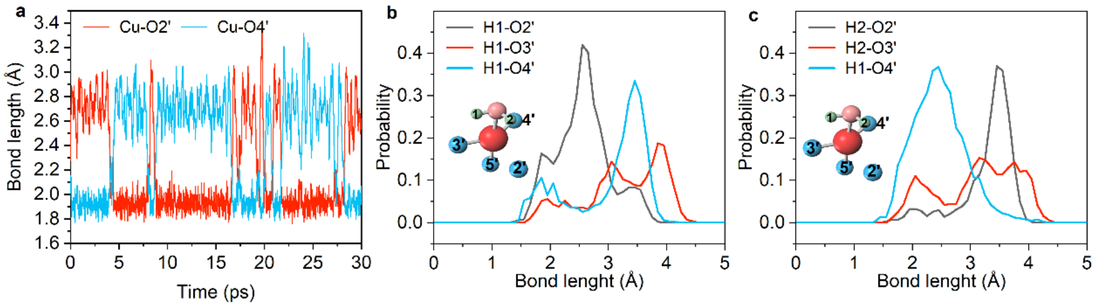

FIG. S8. 30 ps canonical AIMD simulation with the initial temperature of 300 K to verify the local coordination reconstruction of the H_2O adsorption geometry on Cu-SAC/TiO_2. **(a)** Time-dependent Cu-O bond length. **(b, c)** Statistical distributions of the H-O bond length.

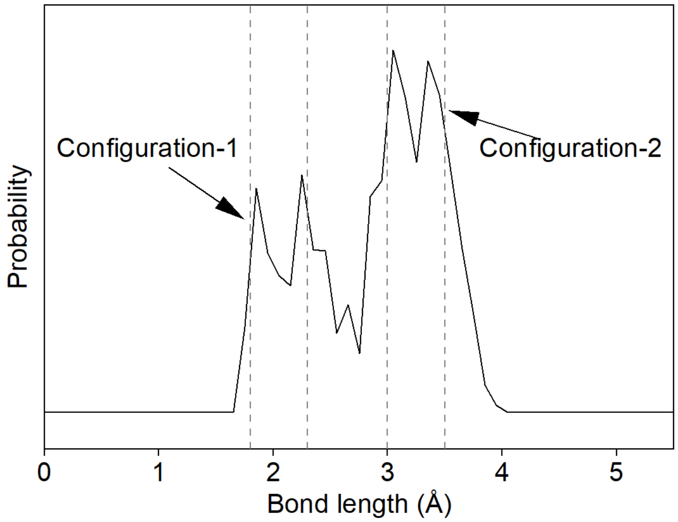

FIG. S9. Statistical distribution of the H1-O3' bond length in the first 3 ps of the canonical AIMD simulation corresponding to Fig. 3b of the main text.

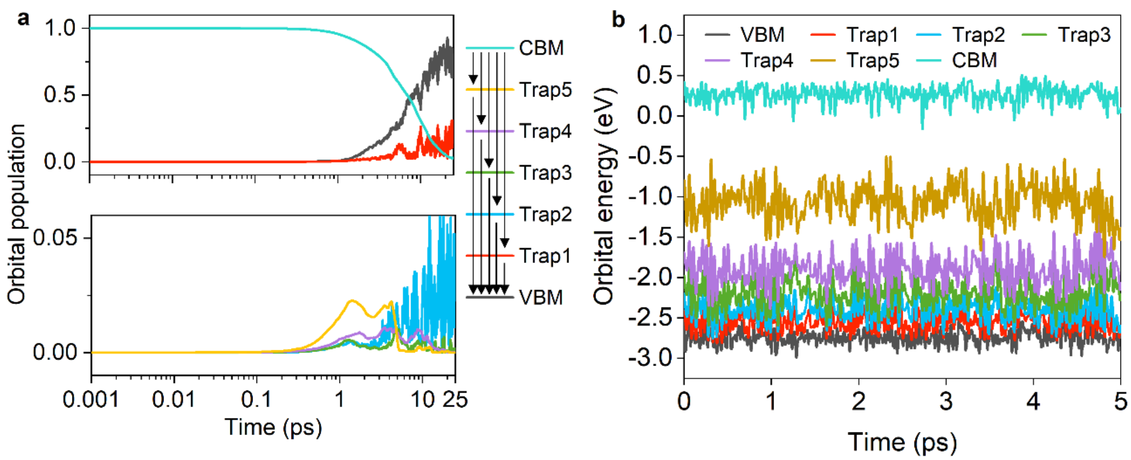

FIG. S10. **(a)** Electron trapping and recombination dynamics in the spin-down channel. **(b)** Evolution of energies of the band edge and trap states for the spin-down channel in Cu-SAC/TiO_2 for the first 5 ps of the microcanonical AIMD trajectory.

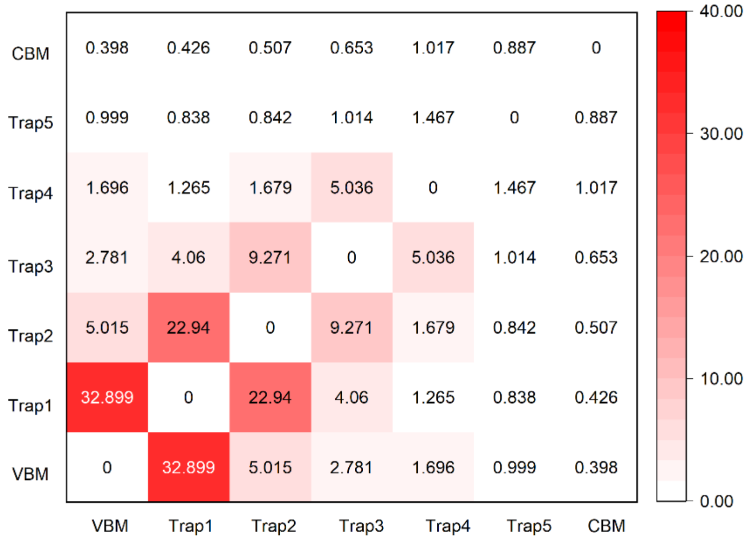

FIG. S11. The average absolute NAC (meV) for Cu-SAC/TiO_2 adsorbed H_2O. NAC between different energy levels affects the transition probability.

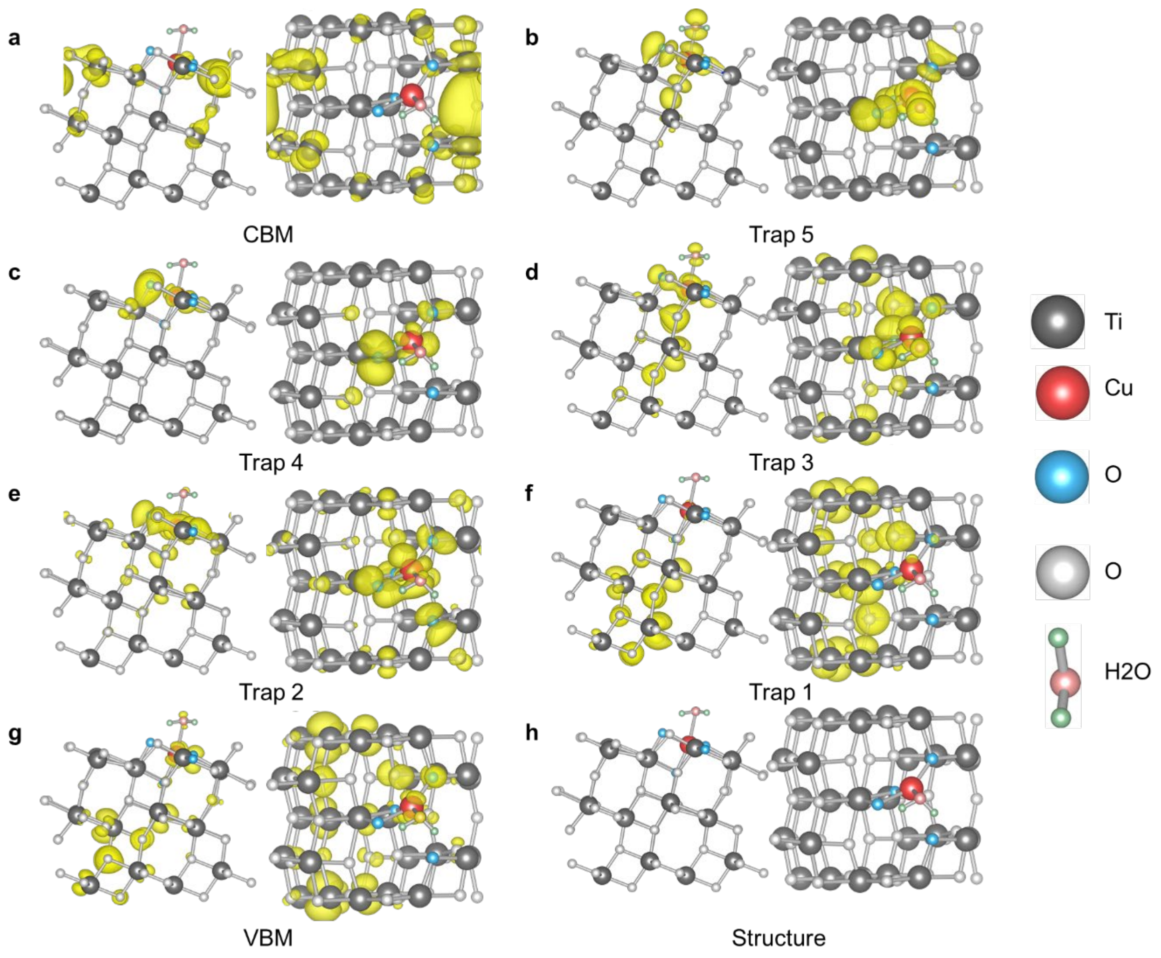

FIG. S12. Spin-down charge densities for VBM, Trap 1-5, and CBM of Cu-SAC/TiO_2.

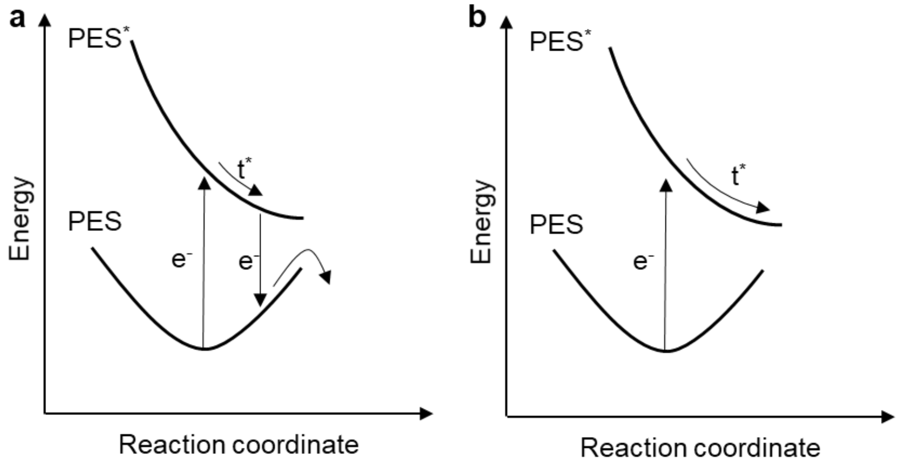

FIG. S13. An illustration of the Impulsive Two-State method.

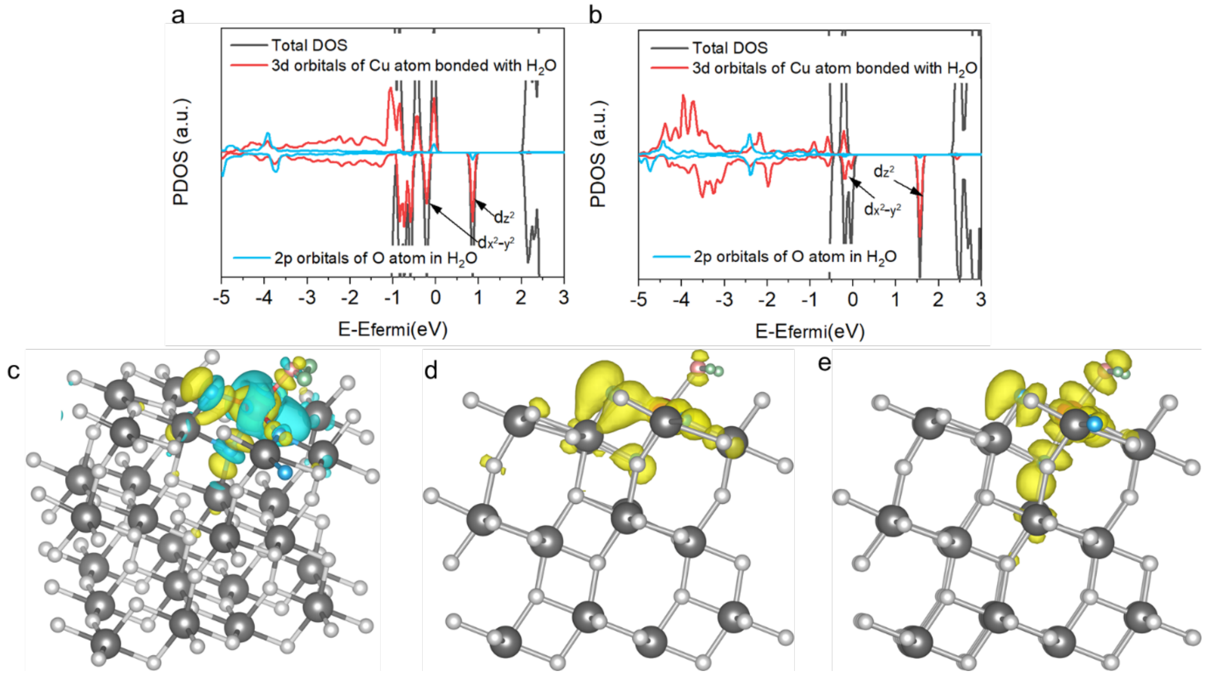

FIG. S14. PDOS of Cu-SAC/TiO_2 with adsorbed H_2O obtained **(a)** without and **(b)** with the +U correction on the Cu atom. U = 4.5 eV is applied to Ti atoms in both parts, while U = 5.2 eV is applied to the Cu atom only in part b. The zero of energy is set to the Fermi level. The energy gaps between the TiO_2 CBM and the d_{x2-y2} and d_{z2} states of Cu undergo minor changes. **(c)** Difference in the total charge density obtained without and with the +U correction on the Cu atom. Charge densities of **(d)** $t_{2g*}(d_{x^2-y^2})$ and **(e)** e_{g*} (d_{z^2}) states obtained with the +U correction on the Cu atom. The Cu d_{x2-y2}/d_{z2} orbitals hybridize with H_2O, similarly to the data in Fig. S11d, e.

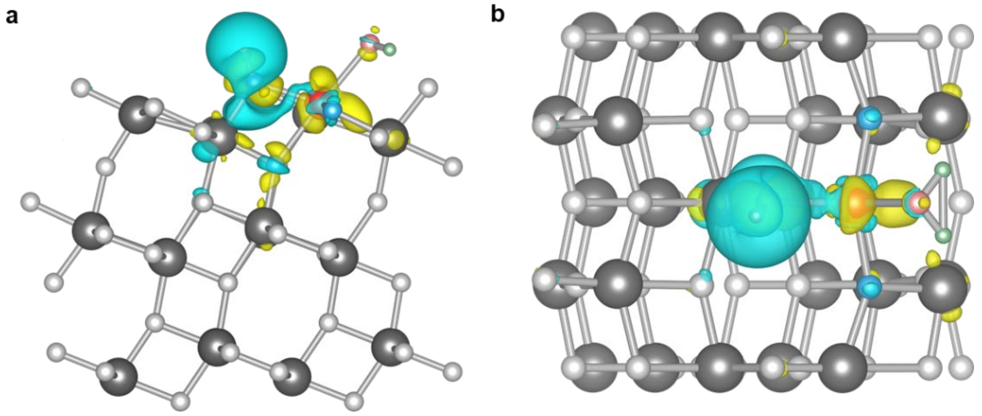

FIG. S15. Charge density difference before and after H adsorption. The extra electron from the H atom is captured by the Cu atom and introduces a repulsion between the Cu atom and the H_2O molecule.

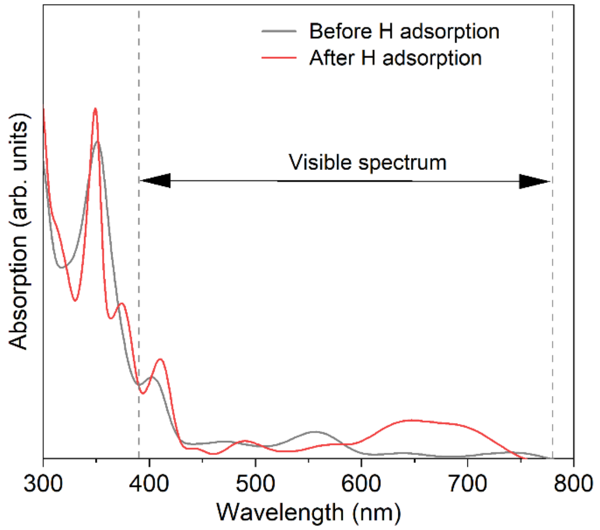

FIG. S16. Absorption spectra of Cu-SAC/TiO_2 before and after H adsorption onto the O atom next to the Cu atom. The calculations are performed with the TDDFT method in Octopus[23].

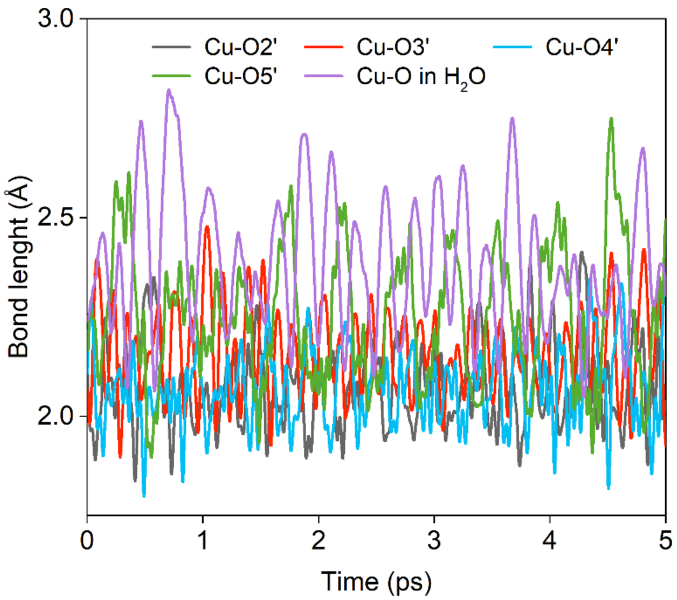

FIG. S17. Time-dependent Cu-O bond length after H adsorption in canonical AIMD with the initial temperature of 300 K.

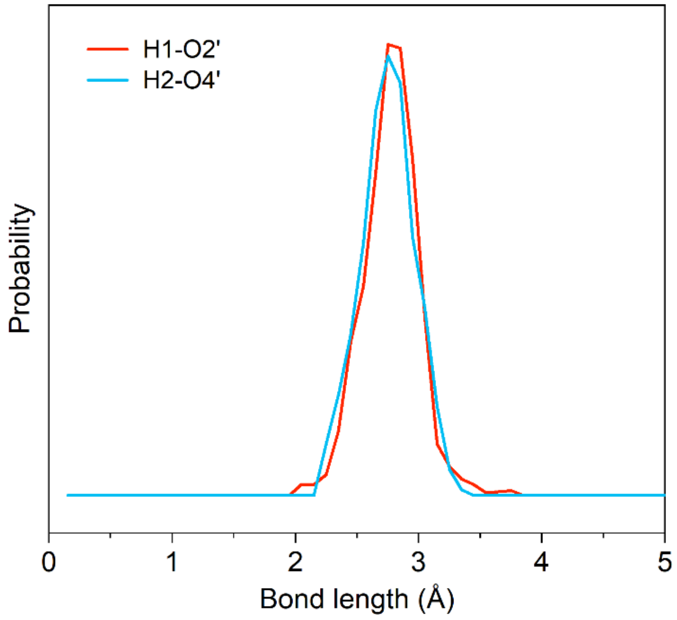

FIG. S18. Statistical distribution of the H-O bond length after H adsorption in canonical AIMD with the initial temperature of 300 K.

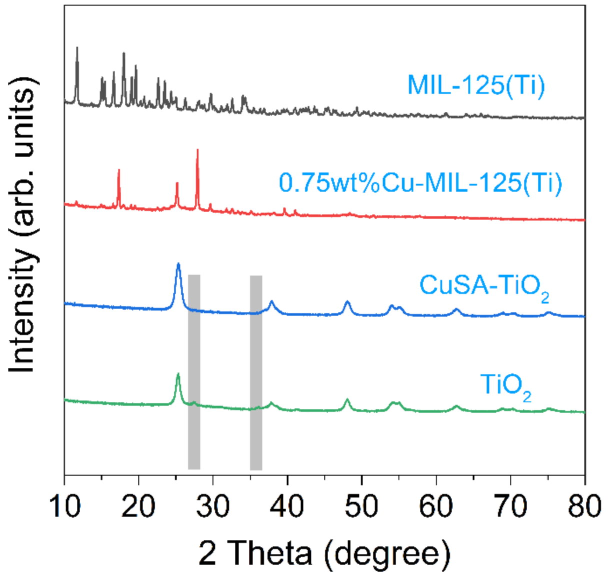

FIG. S19. The XRD images of MIL-125, Cu-MIL-125, TiO_2, and CuSA-TiO_2.

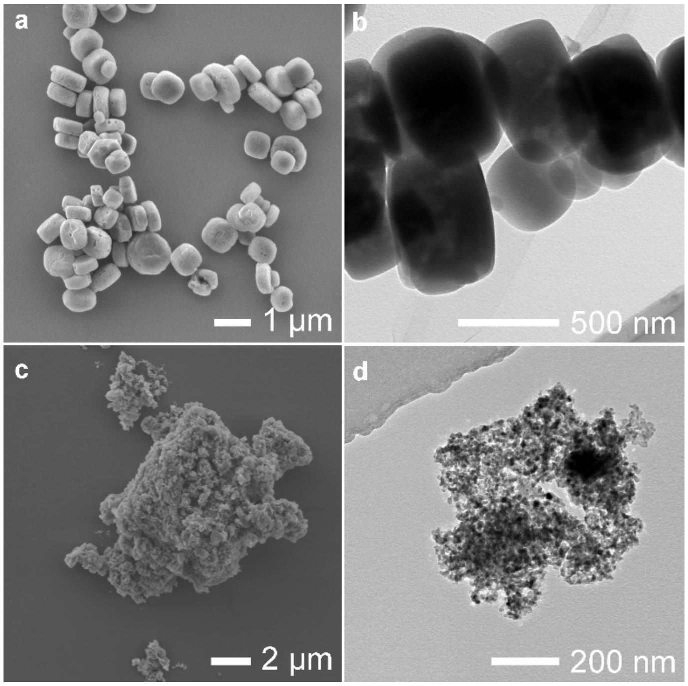

FIG. S20. Representative low **(a)** and high **(b)** magnification SEM images of Cu/TiO_2. Representative low **(c)** and high **(d)** magnification TEM images of Cu/TiO_2.

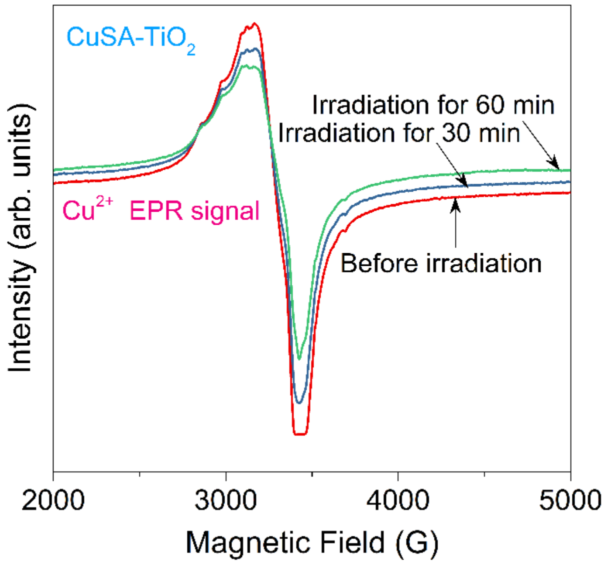

FIG. S21. In-situ electron paramagnetic resonance (EPR) spectra of CuSA-TiO_2 in various states.

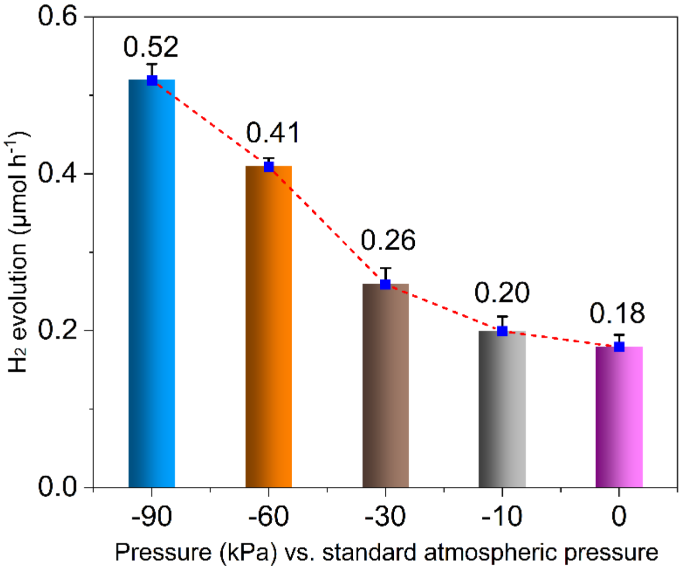

FIG. S22. Photocatalytic hydrogen evolution experiments on Cu-SAC/TiO_2 under different pressures with Ar atmosphere protection. 0 kPa represents the standard atmospheric pressure.

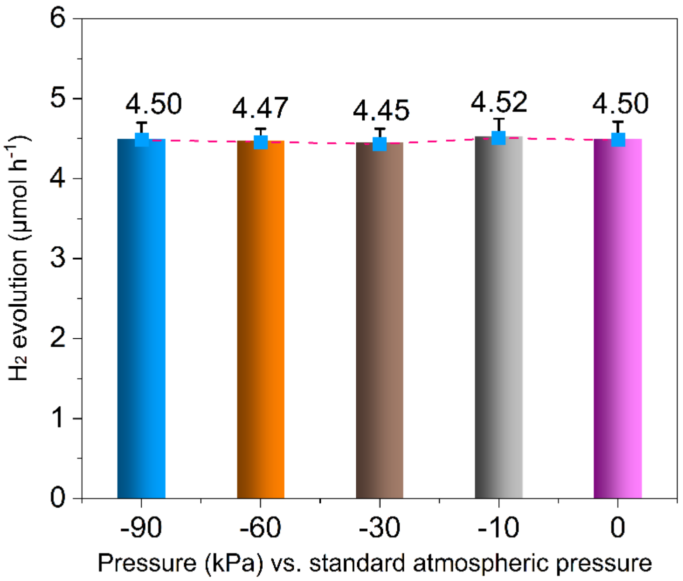

FIG. S23. Photocatalytic hydrogen evolution experiments on pure TiO_2 under different pressures with Ar atmosphere protection. 0 kPa represents the standard atmospheric pressure.

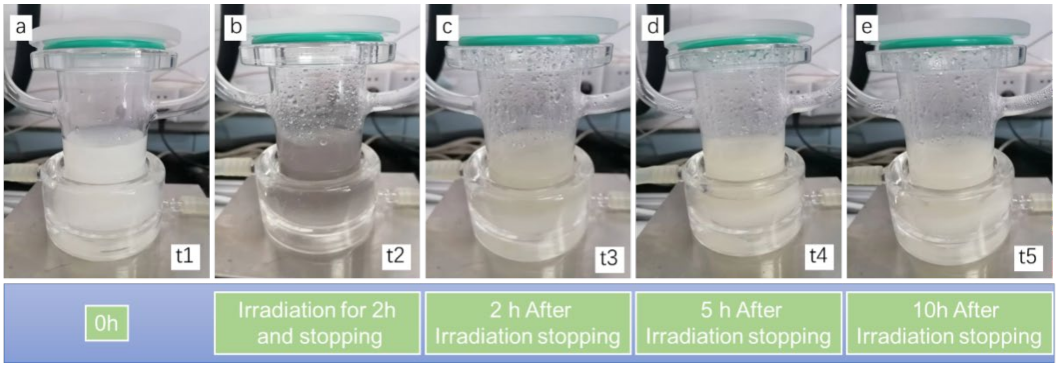

FIG. S24. Reversible color change during and after the photocatalytic reaction. The system is always under Ar atmosphere protection. **(a)-(e)** represent different times during the experiment.

Table S1. Charge population analysis of the Cu atom and its neighboring O atoms

Atom	Mulliken Charge Analysis		Loewdin Charge Analysis	
	Before H adsorption	After H adsorption	Before H adsorption	After H adsorption
O in H_2O	-0.80	-0.84	-0.57	-0.63
O2’	-0.64	-0.63	-0.51	-0.49
O3’	-0.49	-0.74	-0.40	-0.58
O4’	-0.67	-0.63	-0.53	-0.49
O5’	-0.61	-0.49	-0.48	-0.39
Cu	**1.13**	**0.84**	**0.58**	**0.35**

Table S2. ICOHP of the Cu-O bonds before and after H adsorption onto the bridging O atom

Atom-1	Atom-2	Before H adsorption		After H adsorption	
		Spin up	Spin down	Spin up	Spin down
O in H_2O	Cu82	**-1.28**	**-1.50**	**-0.97**	**-0.98**
O2’	Cu82	-0.94	-0.99	-1.51	-1.52
O3’	Cu82	**-1.69**	**-1.94**	**-1.34**	**-1.35**
O4’	Cu82	-1.44	-1.63	-1.50	-1.51
O5’	Cu82	**-1.29**	**-1.74**	**-0.70**	**-0.71**

References

[1] Kresse, G. Ab initio molecular dynamics for liquid metals. J. Non-Cryst. Solids, 1995, 192-193: 222-229.
[2] Kresse, G., Furthmüller, J. Efficiency of ab-initio total energy calculations for metals and semiconductors using a plane-wave basis set. Comput. Mater. Sci., 1996, 6: 15-50.
[3] Kresse, G., Hafner, J. Ab initio hellmann-feynman molecular dynamics for liquid metals. J. Non-Cryst. Solids, 1993, 156-158: 956-960.
[4] Kresse, G., Hafner, J. Ab initio molecular dynamics for liquid metals. Phys. Rev. B, 1993, 47: 558-561.
[5] Blochl, P.E. Projector augmented-wave method. Phys. Rev. B, 1994, 50: 17953-17979.
[6] Kresse, G., Joubert, D. From ultrasoft pseudopotentials to the projector augmented-wave method. Phys. Rev. B, 1999, 59: 1758-1775.
[7] Perdew, J.P., Burke, K., Ernzerhof, M. Generalized gradient approximation made simple. Phys. Rev. Lett., 1996, 77: 3865-3868.
[8] Grimme, S., Antony, J., Ehrlich, S., et al. A consistent and accurate ab initio parametrization of density functional dispersion correction (dft-d) for the 94 elements h-pu. J. Chem. Phys., 2010, 132: 154104.
[9] Kowalski, P.M., Meyer, B., Marx, D. Composition, structure, and stability of the rutile TiO_2 (110)surface: Oxygen depletion, hydroxylation, hydrogen migration, and water adsorption. Phys. Rev. B, 2009, 79: 115410.
[10] Zhang, L., Chu, W., Zhao, C., et al. Dynamics of photoexcited small polarons in transition-metal oxides. J. Phys. Chem. Lett., 2021, 12: 2191-2198.
[11] Cheng, C., Fang, W.H., Long, R., et al. Water splitting with a single-atom cu/tio(2) photocatalyst: Atomistic origin of high efficiency and proposed enhancement by spin selection. JACS Au, 2021, 1: 550-559.
[12] Chu, W., Saidi, W.A., Zheng, Q., et al. Ultrafast dynamics of photongenerated holes at a ch(3)oh/tio(2) rutile interface. J. Am. Chem. Soc., 2016, 138: 13740-13749.
[13] Chu, W., Zheng, Q., Prezhdo, O.V., et al. Co(2) photoreduction on metal oxide surface is driven by transient capture of hot electrons: Ab initio quantum dynamics simulation. J. Am. Chem. Soc., 2020, 142: 3214-3221.
[14] Zheng, Q., Chu, W., Zhao, C., et al. Ab initio nonadiabatic molecular dynamics investigations on the excited carriers in condensed matter systems. Wiley Interdiscip. Rev. Comput. Mol. Sci., 2019, 9: e1411.
[15] Craig, C.F., Duncan, W.R., Prezhdo, O.V. Trajectory surface hopping in the time-dependent kohn-sham approach for electron-nuclear dynamics. Phys. Rev. Lett., 2005, 95: 163001.
[16] Tully, J.C. Molecular dynamics with electronic transitions. J. Chem. Phys., 1990, 93: 1061-1071.
[17] Anggara, K., Huang, K., Leung, L., et al. Bond selectivity in electron-induced reaction due to directed recoil on an anisotropic substrate. Nat. Commun., 2016, 7: 13690.
[18] Huang, K., Leung, L., Lim, T., et al. Single-electron induces double-reaction by charge delocalization. J. Am. Chem. Soc., 2013, 135: 6220-6225.
[19] Leung, L., Lim, T., Ning, Z., et al. Localized reaction at a smooth metal surface: P-diiodobenzene at cu(110). J. Am. Chem. Soc., 2012, 134: 9320-9326.
[20] Li, N., Wang, B., Si, Y., et al. Toward high-value hydrocarbon generation by photocatalytic reduction of co2 in water vapor. ACS Catal., 2019, 9: 5590-5602.
[21] Zhang, Y., Zhao, J., Wang, H., et al. Single-atom cu anchored catalysts for photocatalytic renewable h(2) production with a quantum efficiency of 56. Nat. Commun., 2022, 13: 58.
[22] Lu, K., Hou, F., Fu, W., et al. Efficient solar photocatalytic hydrogen production using direct z-scheme heterojunctions. Phys. Chem. Chem. Phys., 2021, 23: 22743-22749.
[23] Tancogne-Dejean, N., Oliveira, M.J.T., Andrade, X., et al. Octopus, a computational framework for exploring light-driven phenomena and quantum dynamics in extended and finite systems. J. Chem. Phys., 2020, 152: 124119.
